# Primary Role of the Amygdala in Spontaneous Inflammatory Pain- Associated Activation of Pain Networks – A Chemogenetic Manganese-Enhanced MRI Approach

**DOI:** 10.3389/fncir.2019.00058

**Published:** 2019-10-01

**Authors:** Daigo Arimura, Kei Shinohara, Yukari Takahashi, Yae K. Sugimura, Mariko Sugimoto, Tomokazu Tsurugizawa, Keishi Marumo, Fusao Kato

**Affiliations:** ^1^Department of Neuroscience, Jikei University School of Medicine, Tokyo, Japan; ^2^Department of Orthopaedics, Jikei University School of Medicine, Tokyo, Japan; ^3^Center for Neuroscience of Pain, Jikei University School of Medicine, Tokyo, Japan; ^4^NeuroSpin, CEA-Saclay, Gif-sur-Yvette, France

**Keywords:** inflammatory pain, formalin, amygdala, imaging, chemogenetics, MEMRI, DREADD, mice

## Abstract

Chronic pain is a major health problem, affecting 10–30% of the population in developed countries. While chronic pain is defined as “a persistent complaint of pain lasting for more than the usual period for recovery,” recently accumulated lines of evidence based on human brain imaging have revealed that chronic pain is not simply a sustained state of nociception, but rather an allostatic state established through gradually progressing plastic changes in the central nervous system. To visualize the brain activity associated with spontaneously occurring pain during the shift from acute to chronic pain under anesthetic-free conditions, we used manganese-enhanced magnetic resonance imaging (MEMRI) with a 9.4-T scanner to visualize neural activity-dependent accumulation of manganese in the brains of mice with hind paw inflammation. Time-differential analysis between 2- and 6-h after formalin injection to the left hind paw revealed a significantly increased MEMRI signal in various brain areas, including the right insular cortex, right nucleus accumbens, right globus pallidus, bilateral caudate putamen, right primary/secondary somatosensory cortex, bilateral thalamus, right amygdala, bilateral substantial nigra, and left ventral tegmental area. To analyze the role of the right amygdala in these post-formalin MEMRI signals, we repeatedly inhibited right amygdala neurons during this 2–6-h period using the “designer receptors exclusively activated by designer drugs” (DREADD) technique. Pharmacological activation of inhibitory DREADDs expressed in the right amygdala significantly attenuated MEMRI signals in the bilateral infralimbic cortex, bilateral nucleus accumbens, bilateral caudate putamen, right globus pallidus, bilateral ventral tegmental area, and bilateral substantia nigra, suggesting that the inflammatory pain-associated activation of these structures depends on the activity of the right amygdala and DREADD-expressing adjacent structures. In summary, the combined use of DREADD and MEMRI is a promising approach for revealing regions associated with spontaneous pain-associated brain activities and their causal relationships.

## Introduction

Chronic pain is a major health problem, affecting 10–30% of the population in developed countries (Breivik et al., 2006; Hague and Shenker, 2014; Nakamura et al., 2014; Henschke et al., 2015). Although chronic pain is defined as “a persistent complaint of pain lasting for more than the usual period for recovery (usually 3 months)” (Jacobson and Mariano, 2001), recently accumulated lines of evidence obtained by human brain imaging have revealed that chronic pain is not simply a sustained state of nociception, but rather an allostatic state established through plastic and (mal)adaptive changes in the central nervous system (Melzack, 1999; Tracey and Mantyh, 2007; Price et al., 2018). Of a variety of symptoms characterizing chronic pain, an important and the most frequently reported form is “spontaneous” pain, that is, pain perception without specific exogenous painful stimulation, which contrasts it from “evoked” pain. Spontaneous pain is a hallmark of chronic pain symptoms such as those associated with fibromyalgia (Ichesco et al., 2014), arthritis (Kulkarni et al., 2007), the postoperative period (Simanski et al., 2014), and postherpetic neuralgia (Geha et al., 2007). The *de novo* establishment of aberrant brain activity through plastic changes in pain-associated brain regions is proposed to underlie the spontaneous pain (Apkarian et al., 2011; Price et al., 2018). However, despite its devastating impact on the daily life of the patients, the mechanism underlying spontaneous pain in chronic pain patients as well as in animal models of persistent pain remains undetermined.

Some attempts have been made to visualize the brain activity associated with spontaneous pain. Kulkarni et al. (2007) applied positron-emission tomography of the ^18^F-fluorodeoxyglucose uptake to identify the regions specifically activated during the period in which osteoarthritis patients reported spontaneous pain by subtracting the image obtained during the pain-free state from that when the spontaneous pain was being reported. They found that the right amygdala and distinct regions in the cingulate cortex were activated with spontaneous pain. Recently, Hashmi et al. (2013) used a special device enabling the subject to report the subjective detection of spontaneous pain while being scanned in the MRI to identify the brain regions activated during spontaneous pain. They found spontaneous pain-associated activation of the nucleus accumbens and anterior cingulate cortex in patients with a short history of chronic back pain and that of the amygdala and medial prefrontal cortex in patients with a longer history of persistent back pain. Although these pioneering brain imaging studies showed the activation of multiple brain regions during spontaneous pain in human patients, it remains largely unknown which of these structures plays a more primary role in the occurrence of the spontaneous pain-associated brain activation. This question is difficult to address because interventional manipulation of specific brain areas is impossible in human patients. On the other hand, although such interventional manipulation is possible in experimental animal models, objective detection of spontaneous pain in awake, non-anesthetized animals is almost impossible. No strategy has been developed to allow visualization of anesthetic-free activation of spontaneous pain with identification of the causal link between distinct activated brain regions.

Here, we report that the amygdala plays an essential role in the very early period of the pain chronification process in a rodent model of inflammatory pain. To analyze the spontaneous pain-associated brain activities and their causal relationship in the animal models, we applied the following two techniques. First, we used manganese-enhanced magnetic resonance imaging (MEMRI). MEMRI visualizes accumulated manganese (Mn^2+^) through Ca^2+^-permeable channels in excited cells (Silva and Bock, 2008; Kikuta et al., 2015) with T1-weighted MRI (Takeda, 2003; Itoh et al., 2008). In the present study, to evaluate the spontaneous pain-associated regional brain activity in the absence of anesthesia, animals with inflammatory pain were allowed to remain in their home cages for a certain period, during which time Mn^2+^ accumulated in excited brain regions. This enabled *post hoc* identification of the regions that were excited during the observation period by visualizing accumulated Mn^2+^ with a high-magnetic field scanner under deep anesthesia. Second, we used a “chemogenetics” approach to selectively inhibit a set of neurons in a specific region. This technique is based on virus-based transfection of a gene encoding “designer receptors exclusively activated by designer drugs” (DREADD) (Urban and Roth, 2014) into cells in a region of concern. In this study, we used hM4D(G_i_), artificial G protein-coupled receptors that are specifically activated by clozapine-N-oxide (CNO), also an artificial ligand, to attenuate neuronal excitation in cells expressing hM4D(G_i_) receptors in the amygdala. The hypothesis examined was whether activation of the amygdala by sustained nociceptive information in the inflammatory pain model is necessary for the subsequent activation of a subset of brain regions underlying establishment of chronic pain state.

## Materials and Methods

### Animals and Ethical Approval

Male C57BL/6 J mice (7–8 weeks old, weighing 17–24 g) (Sankyo LSC, Inc., CLEA Japan Inc., Tokyo, Japan) were group-housed under a 12-h light/dark cycle and provided with food and water *ad libitum*. The manipulation of the animals was approved by the Institutional Animal Care and Use Committee (IACUC) of the Jikei University and conformed to the Guidelines for Proper Conduct of Animal Experiments of the Science Council of Japan (2006) and to the guidelines of the International Association for the Study of Pain (Zimmermann, 1983).

### General Summary of the Protocols

**FIGURE 1:**
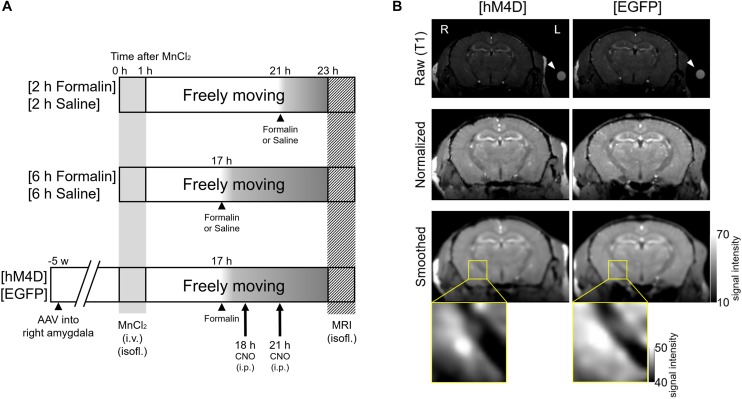
Experimental procedures and visualization of the DREADD effect. **(A)** Mice were divided into three cohorts with two groups each. In the first cohort (Top), formalin or saline was injected 2 h before scanning into two groups of mice, termed [2 h Formalin] (*n* = 8) and [2 h Saline] (*n* = 6), respectively. In the second cohort (Middle), formalin or saline was injected 6 h before scanning, termed [6 h Formalin] (*n* = 7) and [6 h Saline] (*n* = 7), respectively. In the third cohort (Bottom), microinjection of either AAV5-hSyn-HA-hM4D(G_i_)-mCherry or AAV5-hSyn-EGFP into the right amygdala was performed 5 weeks before scanning. These groups were termed [hM4D] (*n* = 6) and [EGFP] (*n* = 6), respectively. Formalin was injected 6 h before scanning and clozapine N-oxide (CNO) was intraperitoneally injected twice at 1 h and 4 h post-formalin. MnCl_2_ solution was injected into all cohorts of mice 23 h before scanning. **(B)** Top, representative coronal T1-weighted raw images of [hM4D] (left) and [EGFP] (right) mouse brains are shown. R, the right side of the mouse; L, the left side. A silicon reference tube filled with 0.1 mM MnCl_2_ solution was attached to the left temporal head of the mouse (arrowhead). Middle, normalized images after registration. Bottom, smoothed images. The statistical analyses described in the text were conducted by using these smoothed images from each mouse. The magnified images on the bottom indicate the signal from the area containing the right amygdala from a mouse from the [EGFP] group and another from the [hM4D] group. Note that the signal is weaker in the central and basolateral amygdala in a [hM4D] mouse.

The mice were divided into three cohorts with different experimental manipulations; each cohort comprised two groups (Figure 1A). In the first cohort, after intraplantar injection of formalin or saline, the mice were allowed to recover in the home cage for 2 h with free access to food and water; at the end of this period, MEMRI was performed according to the procedures described below (Figure 1A). The two groups in this cohort are termed [2 h Formalin] and [2 h Saline] throughout the manuscript and in the figures. In the second cohort, mice spent 6 h in the home cage after intraplantar injection of formalin or saline and then MEMRI was performed (Figure 1A). They are termed [6 h Formalin] and [6 h Saline]. In the third cohort, the mice spent 6 h in the home cage after intraplantar injection of formalin and CNO was intraperitoneally injected twice at 1- and 4 h post-formalin (Figure 1A). The mice in this cohort comprised two groups: mice expressing hM4D(G_i_)-mCherry (called [hM4D]) or EGFP (called [EGFP]) in the amygdala neurons. The gene transfection protocol using adeno-associated vector (AAV) is detailed below. All six groups received intravenous injection of MnCl_2_ solution 23 h before the MRI scanning.

### Viral Vector Injection

The head of a mouse was fixed on a stereotaxic frame (Narishige, Tokyo, Japan). The mouse had first been anesthetized with a mixture of medetomidine hydrochloride (0.3 mg/kg body weight) (Zenoaq, Orion Corporation, Espoo, Finland), midazolam (4.0 mg/kg body weight) (Astellas, Tokyo, Japan), and butorphanol tartrate (5.0 mg/kg body weight) (Meiji Seika Pharma, Tokyo, Japan). The bregma and lambda sutures of the skull were exposed by a midline longitudinal skin incision and pericranial connective tissue removal. Ropivacaine hydrochloride hydrate (7.5 mg/ml; AstraZeneca BV, Zoetermeer, Netherlands) was subcutaneously injected into the skull for local anesthesia. A 500-nl viral solution containing 4.9 × 10^9^ genomic copies/μl of AAV5-hSyn-HA-hM4D(G_i_)-mCherry (Penn Vector Core, Philadelphia, PA, United States) or control virus comprising 4.6 × 10^9^ genomic copies/μl of AAV5-hSyn-EGFP (Penn Vector Core) was injected into the right central amygdala (CeA; 1.20 mm anterior, 2.8 mm lateral, and 4.7 mm ventral from bregma) (Franklin and Paxinos, 2008) using a 30-gauge needle attached to a 2-μl Hamilton syringe controlled by a microinjection pump (UMP-4; World Precision Instruments, Sarasota, FL, United States). The solutions also contained FluoSpheres (1.25%, F-8794 or F-8795, 0.04 μm; Molecular Probes, Thermo Fisher Scientific, Waltham, MA, United States) for the post-experimental verification of injection sites. The mice were allowed to spend 5 weeks in the home cage after the intracranial injection of the viral solutions before the MnCl_2_ injection and subsequent MEMRI.

### Manganese Administration

MnCl_2_ solution was injected into all mouse cohorts 23 h before the MEMRI scan (Figure 1). A solution of 100 mM MnCl_2_ (MnCl_2_⋅4H_2_O; Sigma-Aldrich, St. Louis, MO, United States) was made with distilled water and diluted to 50 mM with saline (osmolarity, 275 mOsm/kg). The MnCl_2_ solution was infused slowly through the tail vein using a syringe pump (KDS-100; KD Scientific, Holliston, MA, United States) for 60 min to a final dose of 75 mg/kg (final volume, 11.92 ml/kg) under continuous anesthesia (0.5–1.5% isoflurane; Mylan Inc., Tokyo, Japan) with monitoring of SpO_2_ with a SomnoSuite (Towa Science, Tokyo, Japan); the mice were placed on a thermoregulated plate to maintain body temperature (Hattori et al., 2013). After MnCl_2_ administration and confirmation of animal recovery from anesthesia, the mice were returned to their home cage and allowed to move freely with free access to food and water until the MRI acquisition. The mice were taken out of the home cage for <1 min for (1) intraplantar injection of formalin or saline (for all three cohorts) and (2) repeated (twice in total) intraperitoneal injections of CNO (for the third cohort). Details of these drug administrations are provided below.

### Intraplantar Injection of Formalin and Evaluation of Nocifensive Behavior

#### Intraplantar Formalin Injection

A 5% formalin solution (37% formaldehyde solution diluted in saline solution) or saline solution (0.9%) was injected (20 μl) into the intraplantar surface of the left hind paw using a microsyringe with a 30-gauge needle (Tjølsen et al., 1992). The timing of the formalin or saline injection differed depending on the cohort group (Figure 1A). The Mn^2+^ enters into the brain tissue through the choroid plexus at which the Mn^2+^ concentration increases immediately after systemic injection of MnCl_2_ and decays to an almost pre-injection level within 2 weeks (Aoki et al., 2004). As the Mn^2+^ concentration in the brain tissue reaches at a steady peak level at 24 h post-MnCl_2_ injection and remains high for a week, it was assumed that the difference between the Mn^2+^ concentrations in the cerebrospinal fluid at 17- and 21 h would be if any limited (Aoki et al., 2004). Acute nocifensive behaviors including licking, lifting, and flinching of the injected paw were visually confirmed in all formalin-injected mice; these behaviors were not seen in saline-injected mice.

#### Nocifensive Behavior Evaluation

In a separate set of animals (24 mice), we examined the effects of MnCl_2_ injection on the acute and latent nocifensive behaviors following formalin injection. MnCl_2_ (75 mg/kg, i.v.; 12 mice) or saline (12 mice) were injected intravenously as described above for the MRI experiments. At 17 h (group 1; 6 mice from MnCl_2_-treated and 6 mice from saline-treated) or 21 h (group 2; 6 from MnCl_2_- and 6 from saline-treated mice), 5% formalin was injected into the intraplantar surface of the left hind paw using a microsyringe with a 30-gauge needle (MnCl_2_- and 6 saline-injected mice for each of groups 1 and 2). The evaluation of nocifensive behaviors was done according to methods described previously (Shinohara et al., 2017; Miyazawa et al., 2018). Briefly, each mouse was put immediately to the observation chamber (17 cm × 17 cm × 35 cm transparent acryl frame) and the recording of spontaneous nocifensive behaviors were then started with a web camera (HD Webcam C525; Logicool, Tokyo, Japan). The time spent performing nocifensive behavior, which was defined by licking the injected paw, was measured with a handheld stopwatch for 10-min intervals up to 1 h after the formalin injection. After the observation of behavior, mice were returned home cage.

For the mice of group 1, we evaluated mechanical threshold of bilateral hind paws using von Frey filaments at 15 h after MnCl_2_ or saline injection (i.e., 2 h before formalin injection) as a baseline and then 6 h after formalin injection. The von Frey filament test was performed according to a previously reported method (Shinohara et al., 2017) by well-trained experimenters who were blinded to the injected solution (MnCl_2_ or saline). Mechanical stimuli were applied with von Frey filaments (North Coast Medical, Gilroy, CA, United States) with different rigidities (0.008–2.0 g). Each mouse was placed on a metal mesh floor (UGO BASILE) and they were allowed to habituate in a 500-mL glass beaker that was placed upside down for 30 min prior to the measurement. A von Frey filament was applied manually from beneath, and the 50% threshold was estimated with the up-and-down method. Care was taken to reduce the number of trials conducted to avoid inflicting unnecessary pain to the animals.

### MRI Acquisitions

The following manipulations for the MRI acquisition were performed 23 h after intravenous administration of MnCl_2_. This timing was selected according to the kinetics of the intracerebral Mn^2+^ concentration (Silva et al., 2004). A mouse was taken from the home cage and anesthetized with 5% isoflurane and fixed in a prone position on the MRI animal heating bed (PN: Z114100; Bruker BioSpin, Ettlingen, Germany). The mouse was continuously anesthetized with 1–2% isoflurane from the admission port of the bed and its head was fixed with a pair of plastic ear bars fixed to a plastic fixing frame. A silicon reference tube (inner diameter, 1 mm; outer diameter, 3 mm; length, 12–15 mm; AS ONE, Tokyo, Japan) was filled with 0.1 mM MnCl_2_ solution and fixed to the skin on the left side of the mouse head with plastic tape to cover the rostrocaudal extent of the MRI scanning for the Mn^2+^ signal intensity reference. The respiratory movement of the chest was monitored with an MRI-compatible transducer (RS-301; Small Animal Instruments, Stony Brook, NY, United States) and the rate was kept at 80–100 breaths/min by adjusting the anesthetic concentration. The rectal temperature was monitored with an MRI-compatible thermosensor (RTP-101-B; Small Animal Instruments) and kept at 37–38°C. Under continuous anesthesia (0.5–1.5% isoflurane), the mouse fixed to the scanning frame was put into a 9.4-T horizontal magnet (BioSpec94/20USR; Bruker BioSpin) with an attached cryogenic quadrature radiofrequency probe (Cryoprobe; Bruker BioSpin). T1-weighted brain images were obtained by a three-dimensional fast low-angle shot (FLASH) sequence with the following parameters: pulse repetition time = 50 ms; echo time = 4.6 ms; flip angle = 30°; number of repetitions = 1; matrix size = 180 × 180 × 100; field of view = 1.8 cm × 1.8 cm × 1.0 cm (100 μm × 100 μm × 100 μm/voxel).

### MRI Data Analysis

After scanning, the T1-weighted images were stored in Bruker format and were converted to ANALYZE format using Bruker2Analyze Paravision Converter^1^ after the experiments. Registration, smoothing, and statistical analysis were performed using SPM8 (Wellcome Department of Cognitive Neurology, London, United Kingdom) running on MATLAB R2014a (MathWorks, Sherborn, MA, United States). The images were registered to the mouse template images, which were created by averaging 20 mouse brain images and co-registered to the Franklin and Paxinos mouse brain atlas (Franklin and Paxinos, 2008). The signal intensities of all images were normalized to the simultaneously measured signal from the reference solution of 0.1 mM MnCl_2_. The images were smoothed with a Gaussian kernel of 0.2 mm full-width at half-maximum. Using a voxel-by-voxel *t*-test for statistical analysis, smoothed images were compared between formalin- and saline-injected animals (i.e., [2 h Formalin] vs. [2 h Saline] and [6 h Formalin] vs. [6 h Saline]), between 2- and 6-h injection animals (i.e., [2 h Saline] vs. [6 h Saline] and [2 h Formalin] vs. [6 h Formalin]), and between [hM4D] and [EGFP]. The comparisons in this study were always made between two datasets with only single difference in the experimental conditions. We did not make any multiple (≥3) group comparisons because it makes the hypothesis to be tested meaningless. The areas showing a significant difference in the manganese signal (*P* < 0.05, corrected for multiple comparisons using the false discovery rate (FDR) procedure at cluster level) were analyzed to construct the T1-weighted images. Average normalized images of every group and statistical maps were displayed with MRIcron^2^. Figure 1B shows two representative images of [hM4D] and [EGFP] that indicate some of the preprocessing stages.

### CNO Agonist Injection

Clozapine-N-oxide (Enzo, Farmingdale, NY, United States) was dissolved in saline (0.9% NaCl). In the third cohort ([hM4D] or [EGFP]), CNO (3 mg/kg intraperitoneal) was administered twice at 1 h and 4 h post-formalin, which are 18- and 21 h post-MnCl_2_, respectively, and at 5- and 3 h before the MRI scanning, respectively (Figure 1A; Coutinho et al., 2017).

### Histological Verification of the Viral Transfection

After the MRI scan, each mouse was removed from the scanner and returned to its home cage. After 1–4 days, each mouse was sacrificed and the whole brain was removed and immersed in a fixative solution (4% paraformaldehyde in phosphate buffer [0.1 M, pH 7.5]) for at least 1 day. Coronal tissue slices were made at a thickness of 100 μm containing the injection sites and hM4D(G_i_)-mCherry– or EGFP–expressing area. The slices were mounted on glass slides and observed using a fluorescent microscope (BX-63; Olympus, Tokyo, Japan) to visualize the expression of mCherry or EGFP. The brain area with mCherry or EGFP expression was depicted by using Photoshop (version 6.1, Adobe Systems, San Jose, CA, United States) as follows: First, epifluorescence brain images were captured at 8-bit resolution with the CCD camera at a resolution of 1.61 μm/pixel, which was then down-sampled to 9.8 μm/pixel to obtain the identical spatial resolution with the brain atlas data. After overlaying the brain image onto the atlas, the orientation and location of the structures were fit on to the atlas. Then, the fluorescence intensity was converted linearly so that the highest intensity of mCherry or EGFP in the image be 100% and the highest non-fluorescent intensity be 0% (in most cases, the hippocampal fimbria was chosen as a site for this). A Gaussian-filter with 10-pixel diameter was applied and the images were binarized at a threshold of 60% (decided empirically) and the borders of the area with signal intensity >60% were drawn with Photoshop. The border curves were overlaid after appropriate compensation of the brain image angle to fit the representative image (e.g., Figures 4B,C).

### *Ex vivo* Electrophysiological Verification of the Effect of CNO in DREADD-Expressing Brain Tissue

Electrophysiological experiments were performed according to previously described procedures from our laboratory (Sugimura et al., 2016; Shinohara et al., 2017). Briefly, 5 weeks after virus injection, the mice were transcardially perfused with ice-cold cutting solution under isoflurane anesthesia (5% in 100% O_2_) and sacrificed. The brain was then removed and a block of the brain containing the amygdala was dissected out and cut at the midline in ice-cold cutting solution composed of (in mM) 2.5 KCl, 0.5 CaCl_2_, 10 MgSO_4_, 1.25 NaH_2_PO_4_, 2 thiourea, 3 sodium pyruvate, 93 *N*-methyl-D-glucamine, 20 HEPES, 12 *N*-acetyl-L-cysteine, 25 D-glucose, 5 L-ascorbic acid, and 30 NaHCO_3_ equilibrated with 95% O_2_ + 5% CO_2_ (osmolality, approximately 290 mOsm/kg). The pH of the solution was titrated to 7.1–7.5 with concentrated HCl. The dissected right hemisphere containing the amygdala was secured on the cutting stage of a vibrating blade slicer (Neo Linear Slicer MT; Dosaka EM) with the rostral end upward to prepare coronal slices (300 μm thick). The slices were first incubated in a holding chamber with a constant flow of cutting solution at 34°C for 15 min. After this initial recovery period, the slices were transferred to another holding chamber containing artificial cerebrospinal fluid (ACSF) composed of (in mM) 125 NaCl, 3 KCl, 2 CaCl_2_, 1.3 MgCl_2_, 1.25 NaH_2_PO_4_, 10 D-glucose, 0.4 L-ascorbic acid, and 25 NaHCO_3_ (pH 7.4 bubbled with 95% O_2_ + 5% CO_2_; osmolality, approximately 310 mOsm/kg) at room temperature (20–25°C) until the electrophysiological recording. Each slice was transferred to a recording chamber (volume, ∼0.4 mL) and fixed with nylon grids to a platinum frame. The slice was submerged in and superfused continuously at a rate of ∼2 mL/min with the ACSF described above.

Neurons in the amygdala were visually identified under an upright microscope (BX51WI; Olympus) with an oblique trans-tissue illumination and epifluorescence observation. Images from slices were captured using a CCD camera [IR-1000 (DAGE-MTI, Michigan City, IN, United States) or Advan Cam-HD1080 (Advan Vision, Tokyo, Japan)] and stored digitally on a computer. Patch-clamp electrodes were made from borosilicate glass pipettes (1B120F-4; World Precision Instruments). The tip resistance of the electrode was 5–8 MΩ. The composition of the internal solution was (in mM) 120 potassium gluconate, 6 NaCl, 1 CaCl_2_, 2 MgCl_2_, 2 ATP Mg, 0.5 GTP Na, 12 phosphocreatine Na_2_, 5 EGTA, and 10 HEPES hemisodium (pH 7.3, adjusted with KOH; osmolality, approximately 290 mOsm/kg). In some recordings, the internal solution contained 0.00125–0.0025% Alexa Fluor 488 or 568. Whole-cell membrane potentials of transfected neurons (identified by mCherry fluorescence) were recorded using an IPA or D-IPA amplifier (Sutter Instrument Company, Novato, CA, United States), filtered at 2 kHz, and digitized at 10 kHz with 16-bit resolution using a PowerLab interface (AD Instruments, Dunedin, New Zealand). After confirmation of the generation of an action potential in response to depolarizing current injections and that the resting membrane potential was less than –45 mV, an appropriate fixed current was injected so that the resting membrane potential was maintained at around –60 mV. This fixed current was not changed during the course of the recording.

In addition, a repeated current command composed of a hyperpolarizing pulse (2000 ms, 20–120 pA) followed by a depolarizing pulse (500 ms, 20–100 pA) was injected every 10 s during the recording. In each neuron, we fixed the amplitude of this hyperpolarizing pulse so that the membrane potential reached around −80 mV and also fixed that of the depolarizing pulse so that it generated approximately around five to ten action potentials (e.g., Figure 5B2 bottom). Once fixed, a stable recording for at least 5 min was obtained (“baseline”), after which CNO was applied for 3 min without alteration of any of these parameters. All recordings were made at room temperature (20–25°C). Only data from the first recording and the first CNO application in each slice were used for analysis. The recorded membrane currents and numbers of action potentials were analyzed offline with IGOR Pro 7 (WaveMetrics, Lake Oswego, OR, United States) using procedures written by Kato et al. (2016).

Values are expressed as the mean ± standard error of the mean. For statistical analysis, the membrane potential values were measured just before and just after the CNO application as “pre” and “CNO,” respectively (e.g., Figure 5C1). The mean number of action potentials appearing during the 500-ms depolarizing steps were calculated from the responses in six cycles just before and just after the CNO application as “pre” and “CNO,” respectively. Differences in the values obtained in electrophysiological recordings were compared using a paired *t*-test, Mann–Whitney *U*-test and Wilcoxon signed rank test. Statistical calculations were made using SPSS 25.0 (IBM, Tokyo, Japan). Differences with *P* < 0.05 were considered significant.

## Results

### Time-Differential MEMRI Analysis Reveals Manganese Accumulation in Corticolimbic Regions in the Late Phase After Formalin Injection

**FIGURE 2:**
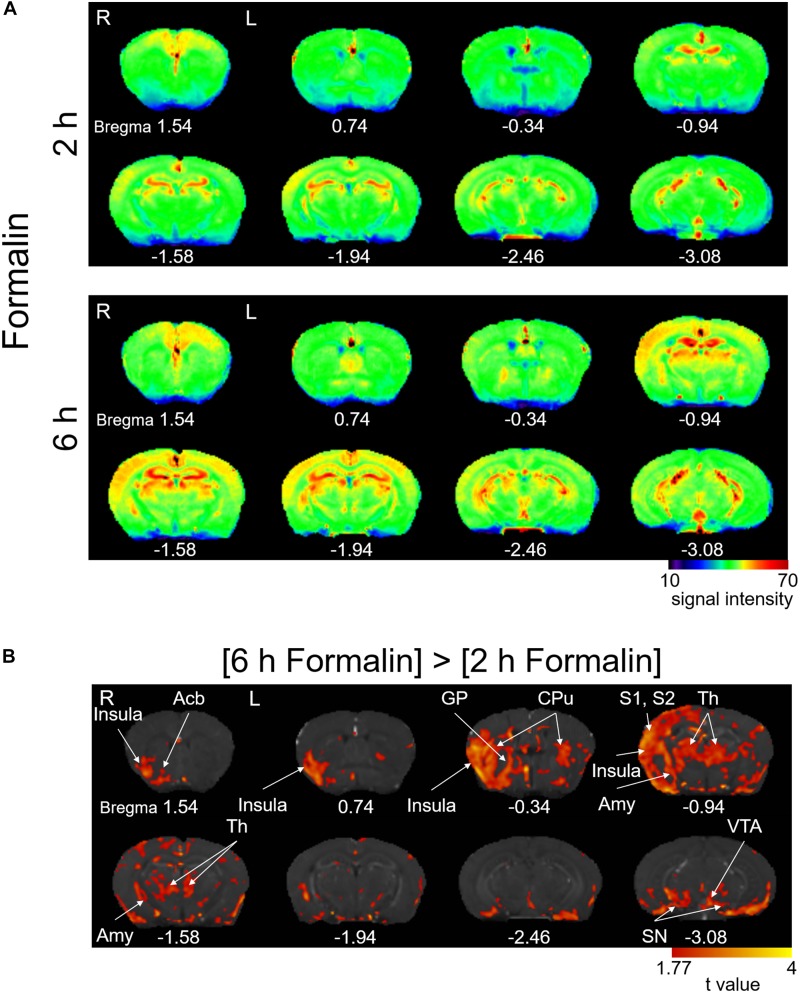
Manganese accumulation in the brain after the freely moving post-formalin period. **(A)** Color map of averaged T1-weighted images in the [2 h Formalin] and [6 h Formalin] groups. R, the right side; L, the left side. The color bar indicates normalized signal intensity. The numbers below the images indicate the anterior-posterior coordinate from bregma. **(B)** Comparisons of the signal intensity between [2 h Formalin] and [6 h Formalin] shown as *t*-value plots (*P* < 0.05, cluster-wise FDR corrected). Insula, insular cortex; Acb, nucleus accumbens; GP, globus pallidus; CPu, caudate putamen; S1/S2, primary/secondary somatosensory cortex; Th, thalamus; Amy, amygdala; SN, substantia nigra; VTA, ventral tegmental area. R, the right side; L, the left side. The color bar indicates the *t*-value.

Intraplantar injection of formalin (5%) into the left hind paw induced acute nocifensive behaviors including licking, lifting, and flinching of the injected paw lasting 60–90 min in all mice injected with formalin (Gong et al., 2014; Shinohara et al., 2017). No animal showed any of these behaviors after saline injection. After this period, the formalin-treated mice did not show any more of these behavioral signs. This formalin-inflammation model shows bilateral tactile allodynia as well as augmented brain expression of phosphorylated extracellular signal-regulated kinase (pERK) 3–6 h after the injection (Carrasquillo and Gereau, 2007; Shinohara et al., 2017), which leads to central plastic changes at 24 h (Adedoyin et al., 2010; Sugimura et al., 2016) and finally neuropathic pain-like phenotypes (Salinas-Abarca et al., 2017). It is thus postulated that the spontaneous brain activity between the initial acute nocifensive phase in the first 90–120 min post-formalin and the subsequent “centralization” phase would represent specific activity linked with the early events of central sensitization in the brain. To visualize the spontaneous brain activity during this centralization phase, we performed brain scanning 2 h post-formalin (Figure 2A, above) and 6 h post-formalin (Figure 2A, below) in separate cohorts and performed a time-differential analysis. The signal intensity of T1-weighted normalized images was generally higher in [6 h Formalin] than in [2 h Formalin] (Figure 2A). The statistical comparison of the T1 signal intensity between [6 h Formalin] and [2 h Formalin] is shown in Figure 2B. The right insular cortex, right nucleus accumbens, right globus pallidus, bilateral caudate putamen, right primary/secondary somatosensory cortex, bilateral thalamus, right amygdala, bilateral substantial nigra, and left ventral tegmental area showed significantly higher intensity in [6 h Formalin] (*P* < 0.05, corrected for multiple comparisons using the FDR procedure at cluster level) (Figure 2B). These structures listed above and in Figure 2B were characterized by large and continuous clusters appearing in the large portion of each structure. However, as seen in Figure 2B, these graphic displays of *t*-value also contain small “blips,” which are difficult to classify into specific functional or anatomical structures. These blips should be interpreted with care because it may reflect significantly higher activities in subsets of neurons within a specific structure or it may have resulted from image processing such as registration. For these reasons, not all the brain structures with significant voxels are shown in the text above and in the Figure 2B. The full results of the time-differential analysis with significant voxels are presented in Table 1.

**TABLE 1:** List of brain regions with voxels showing significantly higher Mn^2+^ signals in [6 h Formalin] than in [2 h Formalin].

**[6 h Formalin] > [2 h Formalin] brain regions**	**Abbreviations in Paxinos atlas**	**Distance from Bregma (mm)**	**Left**	**Right**
Accumbens nucleus	AcbC, AcbSh	1.54	++	+
Caudate putamen (striatum)	CPu			+
Claustrum	CI, DEn, IEn			++
Insular cortex	AIV, AID			+
Piriform cortex	Pir			++
Accumbens nucleus	AcbC, AcbSh	0.74		+
Caudate putamen (striatum)	CPu		+	++
Claustrum	DCl, DEn, IEn, VCl			++
Insular cortex	AIV, AID, DI, GI			++
Lateral septal nucleus	LSI			+
Nucleus of the diagonal band	HDB, VDB			+
Piriform cortex	Pir			++
Somatosensory cortex	S2		+	+
Substantia innominata	SIB			+
Amygdala	ACo, EAC, I	−0.34		++
	AA, CxA, EAM		+	++
Anterior commissure	acp, IPACM, IPACL			++
Basal nucleus (Meynert)	B			++
Bed nucleus of the stria terminalis	st			+
	STMPI		+	+
	STMPL			+
	STMPM		+	+
Caudate putamen (striatum)	CPu		+	++
Claustrum	DCI, DEn, IEn, VCI			++
Globus pallidus	GP, VP		+	++
Hypothalamic nucleus	PaAP, StHy			+
Insula cortex	AIP			++
	DI, GI		+	++
Lateral septal nucleus	LSD			+
Medial forebrain bundle	mfb		+	
Piriform cortex	Pir		+	++
Preoptic nucleus	MCPO, MPA, MPOL			+
Septfimbrial nucleus	SFi		+	+
Somatosensory cortex	S1BF, S1ULp			++
	S2		+	++
Suprachiasmatic nucleus	SCh		+	+
Amygdala	Ast, BLA, CeL, La	−0.94		++
	AA, ACo, BMA, CeC, CeM, CxA		+	++
Anterior commissure	IPAC			++
Anterior hypothalamic area	AHC			+
Caudate putamen (striatum)	CPu		+	+
Claustrum	DEn, VEn, VCI		+	+
Dentate gyrus	DG, MoDG,			+
Globus pallidus	GP		+	++
Hippocampus	fl, Or			++
Insular cortex	DI, GI			++
	AIP		+	+
Laterodorsal thalamic nucleus	LDVL			+
Mammillothalamic tract	mt		+	
Motor cortex	M1		+	+
	M2			+
Optic tract	opt		+	
Piriform cortex	Pir		++	++
Reticular nucleus (prethalamus)	Rt		+	+
Retrochiasmatic area	RCh, RChL		+	+
Retrosplenial cortex	RSD		+	+
	RSGc			+
Somatosensory cortex	S2			++
	S1BF, S1DZ, S1HL, S1Sh, S1ULp		+	++
Stria medullaris	sm		+	+
Supraoptic nucleus	SO			+
Thalamic nucleus	MD, VM		+	
	AD, AM, AV, CM, IAD, IAM, LDVL, PV, Re, Rh, Sub, VA, VL, VPL		+	+
Amygdala	BMA, BLV, PLCo	−1.58	+	++
	ASt, CeL, CeM			+
Caudate putamen (striatum)	CPu			++
Dentate gyrus	DG, GrDG, MoDG, PoDG		+	
Ectorhinal cortex	Ect		+	
Globus pallidus	GP			+
Habenular nucleus	LHb		+	
Hippocampus	CA2			+
Hypothalamic nucleus	VMHC, VMHDM, VMhSh, VMhVL		+	+
Optic tract	opt			+
Parietal association cortex	LPtA			+
	MPtA			+
Medial forebrain bundle	mfb		+	
Nigrostriatal tract	ns		+	
Piriform cortex	Pir		++	++
Retrosplenial cortex	RSD, RSGc		+	+
Reticular nucleus (prethalamus)	Rt		+	+
Somatosensory cortex	S1BF		+	++
	S1Tr, S1ULp			+
Subincertal nucleus	SubI		+	+
Supraoptic decussation	sox			+
Thalamic nucleus	LDVL, VPM, VPL			+
	MDC, MDM, Sub, VM		+	
	CM, LPMR, MDL PC VL		+	+
Zona incerta	ZID, ZIV		+	+
Amygdala	AHiAL, AsSt, LaDL, LaVM,	−1.94		++
Caudate putamen (striatum)	CPu			++
Ectorhinal cortex	Ect		+	+
Entorhinal cortex	DLEnt		+	+
Hypothalamic nucleus	DMV		+	+
Medial tuberal nucleus	MTu		+	+
Perifornical nucleus	PeF		+	+
Piriform cortex	Pir		++	
Retrosplenial cortex	RSD		+	
	RSGc		+	+
Somatosensory cortex	S1BF		+	
Temporal association cortex	TeA		+	
Thalamic nucleus	VPL, MDM		+	
Amygdala	AHiAL, AHiPM, PMCO, PLCO	−2.46	++	++
	RAPir			+
Dorsolateral entorhinal cortex	DLEnt			
Ectorhinal cortex	Ect		+	
Hypothalamic nucleus	PH		+	+
Piriform cortex	Pir		+	
Temporal association cortex	TeA		+	
Amygdala	AHiPM, APir, BLP	−3.08	++	++
Anterior pretectal nucleus	APT		+	
Ectorhinal cortex	Ect		+	
Entorhinal cortex	DIEnt, DLEnt		+	+
Hippocampus	AIV, ml, Or			+
Interpeduncular fossa	IPF		+	+
Nucleus of darkschewitsch	Dk			+
P1 reticular formation	p1Rt		+	
Peripeduncular nucleus	PP			+
Perirhinal cortex	PRh		+	
Retroethmoid nucleus	REth		+	+
Substantia nigra	SNL			+
	SNR		+	+
Superior cerebellar peduncle	scp		+	
Temporal association cortex	TeA		+	
Thalamic nucleus	SPFPC, VLi			+
	PIL		+	
Ventral tegmental area	VTA		+	+

*The regions were identified by overlaying the Franklin and Paxinos mouse brain atlas (Franklin and Paxinos, 2008) on the *t*-value plots (Figure 2B), which had been registered onto the same Franklin and Paxinos atlas at the corresponding anterior-posterior level (Supplementary Figures S1_1A–8A). Abbreviations follow those used in the atlas. Figures indicate the distance from bregma (mm). +, *p* < 0.05; ++, *p* < 0.025.*

### DREADD-Induced Reduction of Manganese Accumulation in Various Brain Areas

**FIGURE 3:**
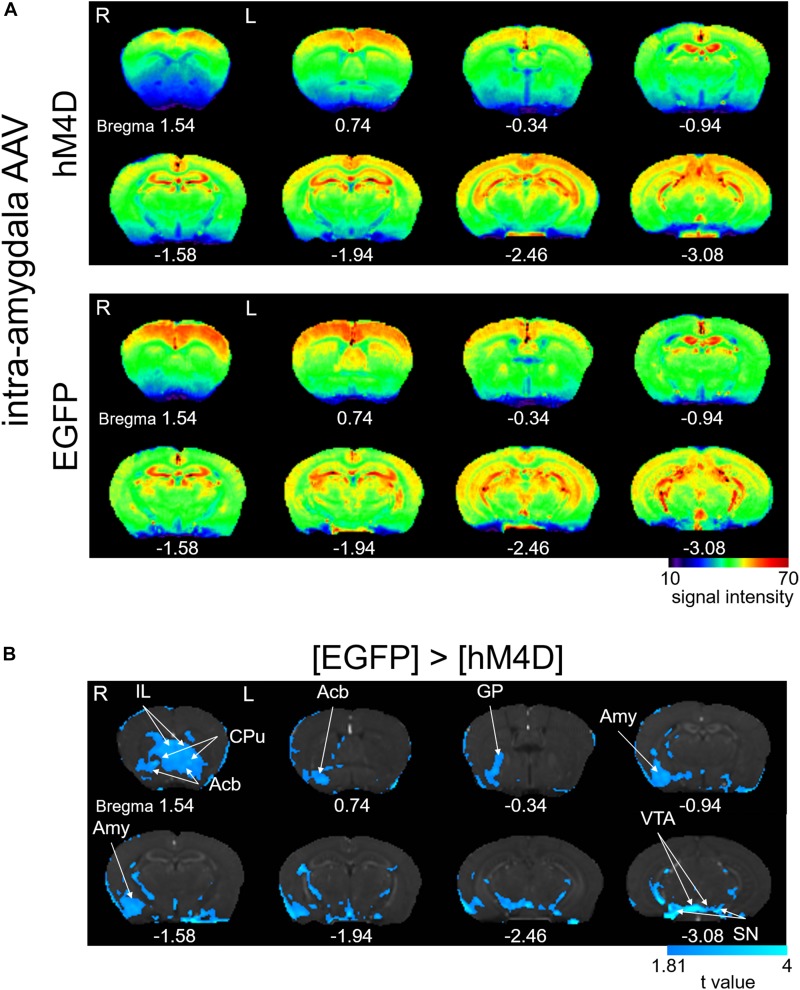
Effects of repeated activation of hM4D receptors expressed in the right amygdala on manganese accumulation. **(A)** Color map display of averaged T1-weighted images in [hM4D] and [EGFP]. R, the right side; L, the left side (for all panels in this figure). The color bar indicates normalized signal intensity. The numbers below the images indicate the anterior-posterior coordinate from bregma. **(B)** Comparisons of the signal intensity between [hM4D] and [EGFP] shown as *t*-value plots (*P* < 0.05, cluster-wise FDR corrected). IL, infralimbic cortex; Acb, nucleus accumbens; GP, globus pallidus; Amy, amygdala; VTA, ventral tegmental area; SN, substantia nigra. The color bar indicates the *t*-value (the signals with [hM4D] were significantly smaller than those with [EGFP]).

In human patients and animal models of chronic pain, the interconnection between distinct concurrently activated brain areas plays essential roles (Baliki et al., 2014), which provide the basis for the network theory of chronic pain state establishment. Formalin injection results in a selective increase in pERK and c-Fos expression as well as synaptic potentiation in the right CeA, which is accompanied by ectopic sensitization in the non-inflamed hind paw at 3–6 h post-formalin (Carrasquillo and Gereau, 2007; Shinohara et al., 2017; Miyazawa et al., 2018). In addition, pharmacological inhibition of the ERK signaling cascade in the right CeA following intraplantar formalin injection suppresses this bilateral sensitization (Carrasquillo and Gereau, 2007). A recent study indicated that optogenetic activation of the right CeA, but not that of the left amygdala, increases bladder nociception (Sadler et al., 2016). Therefore, we examined how the pattern of brain activation observed after formalin injection is affected by repeated selective inhibition of the right amygdala. The average signal intensity was lower in [hM4D] than in [EGFP] in many regions that received repeated CNO injections (Figure 3A). The difference in T1 signal intensity was statistically compared between [hM4D] and [EGFP] (Figure 3B). In addition to the manifest and significant signal decrease in the right amygdala expressing hM4D receptors, the following regions also showed significant decrease in signal intensity: the bilateral infralimbic cortex, bilateral nucleus accumbens, bilateral caudate putamen, right globus pallidus, bilateral ventral tegmental area, and bilateral substantia nigra (*P* < 0.05; corrected for multiple comparisons using the FDR procedure at cluster level) (Figure 3B). Interestingly, no regions showed a significantly higher manganese signal in [hM4D] than in [EGFP] (*P* < 0.05, corrected for multiple comparisons using the FDR procedure at cluster level). The detailed results of the [hM4D]–[EGFP] comparisons are presented in Table 2 and Supplementary Figures S1_8B.

**TABLE 2:** List of brain regions with voxels showing significantly lower Mn^2+^ signals in [hM4D] than in [EGFP].

**[EGFP] > [hM4D] Brain regions**	**Abbreviations in Paxinos atlas**	**Distance from Bregma (mm)**	**Left**	**Right**
Accumbens nucleus	AcbC, AcbSh	1.54	++	+
Caudate putamen (striatum)	CPu		++	++
Claustrum	DEn, IEn		+	+
Dorsal peduncular nucleus	DP		++	++
Dorsal tenia tecta	DTT		++	+
Hippocampus	SHi		+	+
Inflalimbic cortex	IL		++	++
Lateral septal nucleus	LSl		+	+
Accumbens nucleus	AcbC, AcbSh	0.74		+
Caudate putamen (striatum)	CPu			+
Claustrum	IEn			+
Insular cortex	AID, DI			+
Lateral stripe of the striatum	LSS			+
Piriform cortex	Pir			+
Somatosensory cortex	S2			+
Amygdala	I, AA	−0.34		+
Anterior commissure	acp			+
Claustrum	VEn			+
Caudate putamen (striatum)	CPu			+
Globus pallidus	GP			+
Insula cortex	AIP			+
Interstitial nucleus	IPACL			+
Piriform cortex	Pir			+
Amygdala	AA, BLA, BMA, CeC, CxA, IM, La, MeAD	−0.94		++
Caudate putamen (striatum)	CPu			++
Claustrum	DEn, VEn			++
Globus pallidus	GP			+
Hypothalamic nucleus	PaLM, PaMP, PLH			+
Piriform cortex	Pir			+
Amygdala	ASt, BLA, BLP, BLV, CeC, CeL, CeM, I, LaDL, LaVL, PLCo	−1.58		++
	BMA, MePD, MePV		++	++
Bed nucleus of the stria terminalis	STIA			+
Claustrum	DEn, VEn			+
Hypothalamic nucleus	VMHVL, VMHSh		+	
	VMHC		+	+
Medial forebrain bundle	mfb		+	
Piriform cortex	Pir			++
Thalamic nucleus	Xi		+	+
Amygdala	BLP, BLV, BMP, MePD	−1.94		++
	MePV		++	
Bed nucleus of the stria terminalis	STIA			+
Cerebral peduncle	cp			+
Claustrum	DEn, VEn			++
Entorhinal cortex	DLEnt			+
Hippocampus Hypothalamic nucleus	fi			+
	Te			+
	DMC, DMD, DMV, PHD, Pe		+	+
Medial tuberal nucleus	MTu		+	+
Piriform cortex	Pir			+
Reticular nucleus (prethalamus)	Rt		+	+
Subthalamic nucleus	STh		+	+
Zona incerta	ZID			+
Amygdala	BLP	−2.46		+
	AHiPM, PLCo, PMCo		++	
Cerebral peduncle	cp		+	+
Claustrum	DEn, VEn			+
Dentate gyrus	DG		+	
Dorsal lateral geniculate nucleus	DLG		+	
Hippocampus	Py		+	
Hypothalamic nucleus	Gem, PH		++	++
Intergeniculate leaflet	IGL		+	
Nigrostriatal tract	ns		+	++
Piriform cortex	Pir			++
Pregeniculate nucleus	PGMC		+	
Substantia nigra	SNR			++
Subthalamic nucleus	PSTh		+	+
Zona incerta	ZIV		+	++
Cerebral peduncle	cp	−3.08	+	+
Dentate gyrus	DG		+	+
Entorhinal cortex	DLEnt			++
Hippocampus	CA3, Rad, SLu			+
Interfascicular nucleus	IF		++	++
Interpeduncular fossa	IPF		++	++
Interstitial nucleus of Cajal	InC			+
Lateral terminal nucleus	LT		+	
Mammillary peduncle	mp		++	++
Medial geniculate nucleus	MGV		+	
Medial terminal nucleus	MT		+	+
Nucleus of darkschewitsch	Dk			+
Peripedunclar nucleus	PP		+	
Retromammillary nucleus	RM		++	++
Substantia nigra	SNCM, SNR		++	++
Thalamic nucleus	PIL		+	
Ventral tegmental area	PBP, PN, VTA		+	++
Zona incerta	ZIC		+	

*The regions were identified by overlaying the Franklin and Paxinos mouse brain atlas (Franklin and Paxinos, 2008) on the *t*-value plots (Figure 3B), which had been registered onto the same Franklin and Paxinos atlas at the corresponding anterior-posterior level (Supplementary Figures S1_1A–8A). Abbreviations follow those used in the atlas. Figures indicate the distance from bregma (mm). +, *p* < 0.05; ++, *p* < 0.025.*

**FIGURE 4:**
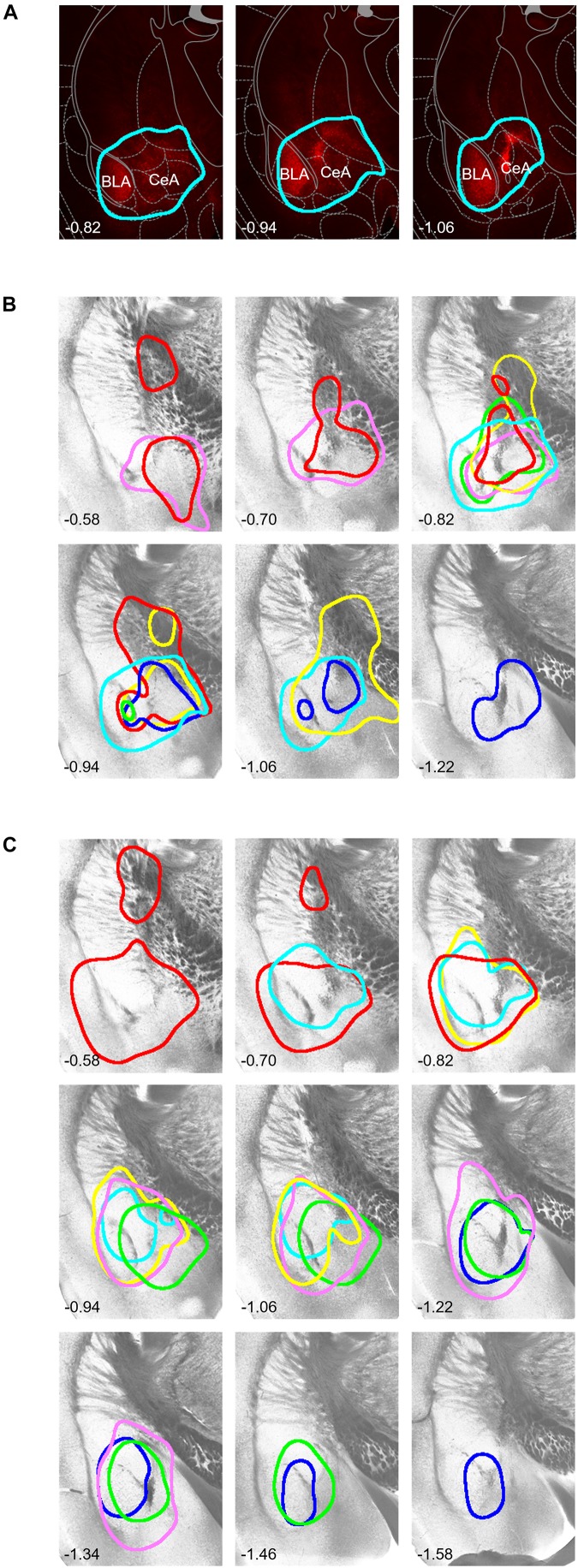
Histological identification of the area with DREADD expression. **(A)** Representative examples of mCherry fluorescence visualized 5 weeks after local injection of hM4D(G_i_)-mCherry–expressing viral solution into the right amygdala. The numbers on the bottom-left show the anterior-posterior level from bregma [also for **(B,C)**]. The expression area is surrounded by a light blue line (see section “Materials and Methods” for the area thresholding with mCherry signals). **(B)** Borders of the mCherry fluorescence in each mouse on representative tissue micrographs from a mouse. Borders drawn with the same color indicate those from the same mouse. **(C)** Borders of the EGFP fluorescence. Note that the fluorescence signals covered most regions of the central and basolateral amygdala. The drawings and schema in panel **(A)** are partially taken from the atlas by Franklin and Paxinos (2008), used with permission.

After the MEMRI scanning, we examined each mouse’s brain and confirmed the regions of hM4D and EGFP (Figure 4). The high-intensity mCherry expression was mostly limited to the central and basolateral amygdala (BLA) and occasionally in the globus pallidus located immediately dorsal to the CeA, presumably due to the leak of virus vector solution during pipette descend toward the CeA. These structures are called “the amygdala and transfected structures” hereafter. The data of two mice (one from [hM4D] and another from [EGFP]) were excluded from the MEMRI analysis because the expression of mCherry and EGFP was too weak to confirm their distribution.

**FIGURE 5:**
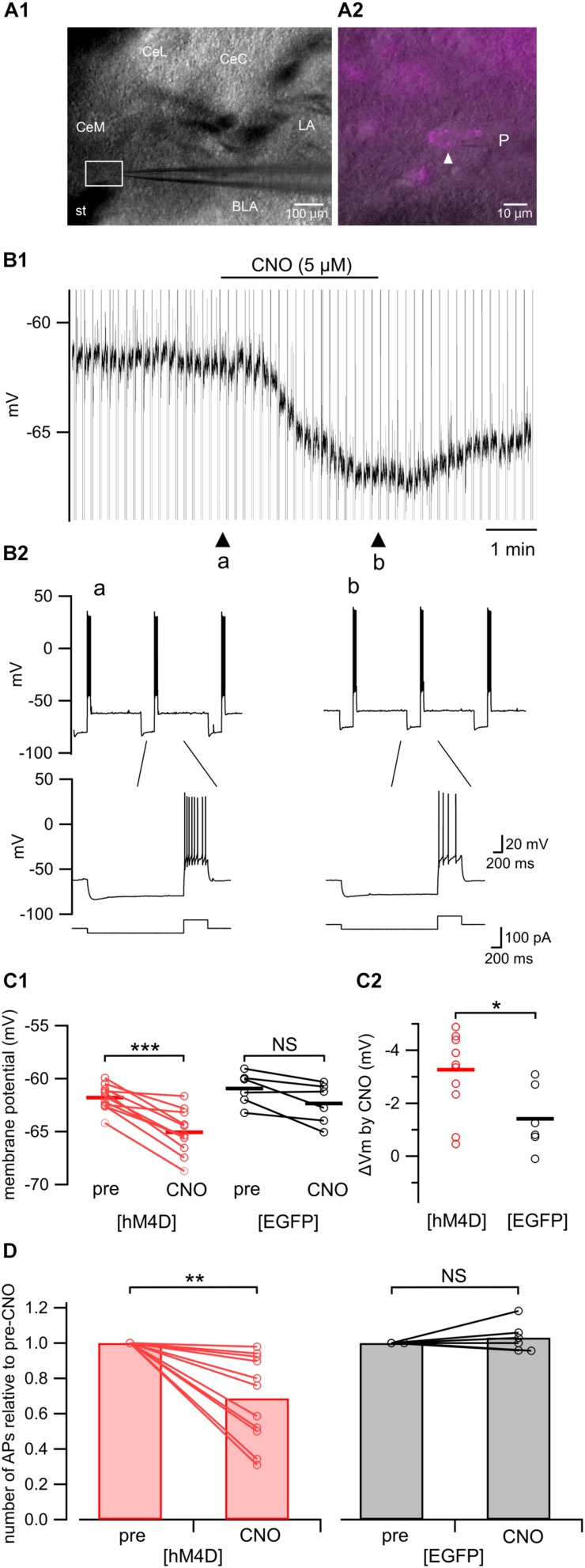
Electrophysiological recordings from hM4D(G_i_)-mCherry-expressing amygdala neurons in acute slices. **(A1)** A representative microphotograph of the coronal amygdala slice used for patch-clamp recording. CeM, medial subdivision of the central amygdala; CeL, lateral subdivision of the central amygdala; CeC, capsular subdivision of the central amygdala; LA, lateral amygdala; BLA, basolateral amygdala; st, stria terminalis; P, recording pipette. **(A2)** A magnified image of the boxed area in **A1**. A white arrowhead indicates a recorded neuron expressing hM4D(G_i_)-mCherry. P, recording pipette. **(B1)** A representative trace of the patch-clamp recording from the hM4D(G_i_)-mCherry-positive neuron shown in **A2** in current clamp mode. CNO (5 μM) hyperpolarized this neuron. **(B2)** Upper, representative responses recorded from a hM4D(G_i_)-positive neuron in the amygdala to a current step (–20 pA, 2 s and 60 pA, 0.5 s, shown in the bottom of **B2**) before (arrowhead, “a” in **B1**) and after (arrowhead, “b” in **B1**) CNO (5 μM) application. Lower, time-extended version of the above traces. **(C1)** Summary of the membrane potential before (pre) and after (CNO) CNO (5 μM) application in hM4D(G_i_)-mCherry–expressing (red; left, *n* = 11) and EGFP-expressing (black; right, *n* = 6) neurons. The horizontal lines indicate the averages. ^∗∗∗^*P* < 0.0001; NS, not significantly different; paired *t*-test. **(C2)** Summary of the CNO (5 μM)-induced change in the membrane potential in hM4D(G_i_)-mCherry–expressing (red, left, *n* = 11) and EGFP-expressing (black, right, *n* = 6) neurons. The horizontal lines indicate the group averages. ^∗^*P* < 0.05; Mann–Whitney *U*-test. **(D)** Summary of the mean number of action potentials relative to the pre-CNO value appearing during the 500-ms depolarizing step before (pre) and after (CNO) CNO (5 μM) application in hM4D(G_i_)-mCherry–expressing (left, *n* = 11) and EGFP-expressing (right, *n* = 6) neurons. ^∗∗^*P* < 0.005; NS, not significantly different; Wilcoxon signed-rank test.

We confirmed that application of CNO indeed inhibited the excitation of neurons expressing hM4D receptors in the amygdala (Figure 5). Figure 5B shows the representative effects of CNO (5 μM) on a CeA neuron expressing mCherry prepared from a [hM4D] mouse (Figure 5A2); CNO hyperpolarized the neuron (Figure 5B1) and decreased the number of action potentials in response to a depolarizing pulse (Figure 5B2). This hyperpolarization with CNO was significant in neurons from [hM4D] mice (Figure 5C1, *P* < 0.0001, paired *t*-test; before CNO [“pre”], –61.8 ± 0.3 mV; after CNO [“CNO”], –65.1 ± 0.6 mV), but not in neurons from [EGFP] mice (Figure 5C1, NS, paired *t*-test; before CNO [“pre”], –61.0 ± 0.6 mV; after CNO [“CNO”], −62.4 ± 0.7 mV). The hyperpolarization induced by CNO in neurons from [hM4D] mice was significantly larger than that in neurons from [EGFP] mice (Figure 5C2, *P* < 0.05, Mann–Whitney *U*-test). In addition, CNO significantly reduced the number of action potentials relative to pre-CNO from [hM4D] mice (Figure 5D, *P* < 0.005, Wilcoxon signed-rank test; from 6.1 ± 0.4 to 4.2 ± 0.5 action potentials/500 ms; *n* = 11) but not in neurons from [EGFP] mice (Figure 5D, NS; from 4.8 ± 0.4 to 5.0 ± 0.5 action potentials/500 ms; *n* = 6).

### Effect of Saline Injection on the Manganese Signal at 2 and 6 h

**FIGURE 6:**
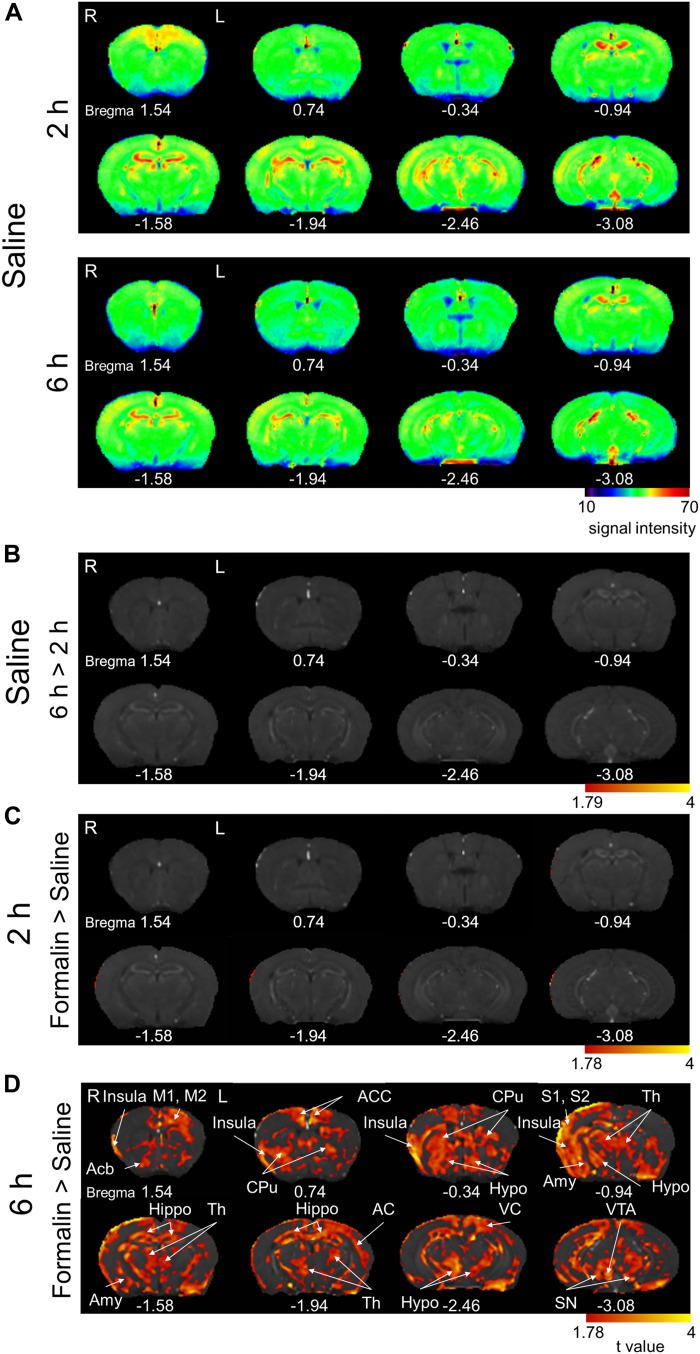
Comparison of the manganese accumulation in the brain between formalin-treated and saline-treated groups. **(A)** Color map display of averaged T1-weighted images in the [2 h Saline] and [6 h Saline] groups. R, the right side; L, the left side (for all panels in this figure). The numbers below the images indicate the anterior-posterior coordinate from bregma. The color bar represents the normalized signal intensity. **(B)** Statistical comparisons of the signal intensity between [2 h Saline] and [6 h Saline] (*P* < 0.05, cluster-wise FDR corrected). The color bar indicates the *t*-value. **(C)** Comparisons of the signal intensity between [2 h Formalin] and [2 h Saline] shown as *t*-value plots (*P* < 0.05, cluster-wise FDR corrected). The color bar indicates the *t*-value. **(D)** Comparisons of the signal intensity between [6 h Formalin] and [6 h Saline] shown as *t*-value plots (*P* < 0.05, cluster-wise FDR corrected). Insula, insular cortex; Acb, nucleus accumbens; M1/2, primary/secondary motor cortex; ACC, anterior cingulate cortex; CPu, caudate putamen; S1/2, primary/secondary somatosensory cortex; Th, thalamus; Amy, Amygdala; Hippo, Hippocampus; AC, auditory cortex; Hypo, hypothalamus; VC, visual cortex; VTA, ventral tegmental area; SN, substantia nigra. R, the right side; L, the left side. The color bar shows the *t*-value.

We observed regions with Mn^2+^ accumulation in some brain structures at 2- and 6 h post-saline (Figure 6A). However, the intensity was generally not stronger at 6 h than at 2 h, unlike after formalin injection (Figure 2A). Indeed, we failed to find regions showing a significantly stronger signal at 6 h than at 2 h post-saline (Figure 6B). Unexpectedly, we also failed to find any brain regions showing a significant difference between formalin- and saline-injected groups at 2 h (Figure 6C) (*P* < 0.05, corrected for multiple comparisons using the FDR procedure at cluster level), suggesting that the accumulation of Mn^2+^ in neurons in the first 2 h is not high enough to exceed the significance level even in the formalin-treated animals, primarily due to the shorter period for accumulation and secondarily due to reduced, if any, Mn^2+^ concentration in the cerebrospinal fluid at 21 h post Mn^2+^ injection than at 17 h. Significant activation was observed in widely distributed brain regions only when compared between formalin and saline injections at 6 h (Figure 6D). Compared with [6 h Saline] (*P* < 0.05, corrected for multiple comparisons using the FDR procedure at cluster level), the activated regions included the right insula cortex, right nucleus accumbens, left primary/secondary motor cortex, bilateral anterior cingulate cortex, bilateral caudate putamen, right primary/secondary somatosensory cortex, bilateral thalamus, right amygdala, bilateral hippocampus, left auditory cortex, bilateral hypothalamus, left visual cortex, right ventral tegmental area, and bilateral substantia nigra. These results suggest that the brain activation during the early nocifensive phase after formalin injection is limited, whereas that during the chronification process between 2- and 6 h post-formalin is potent and widely distributed in the brain.

### Behavioral Consequences of MnCl_2_ Injection

**FIGURE 7:**
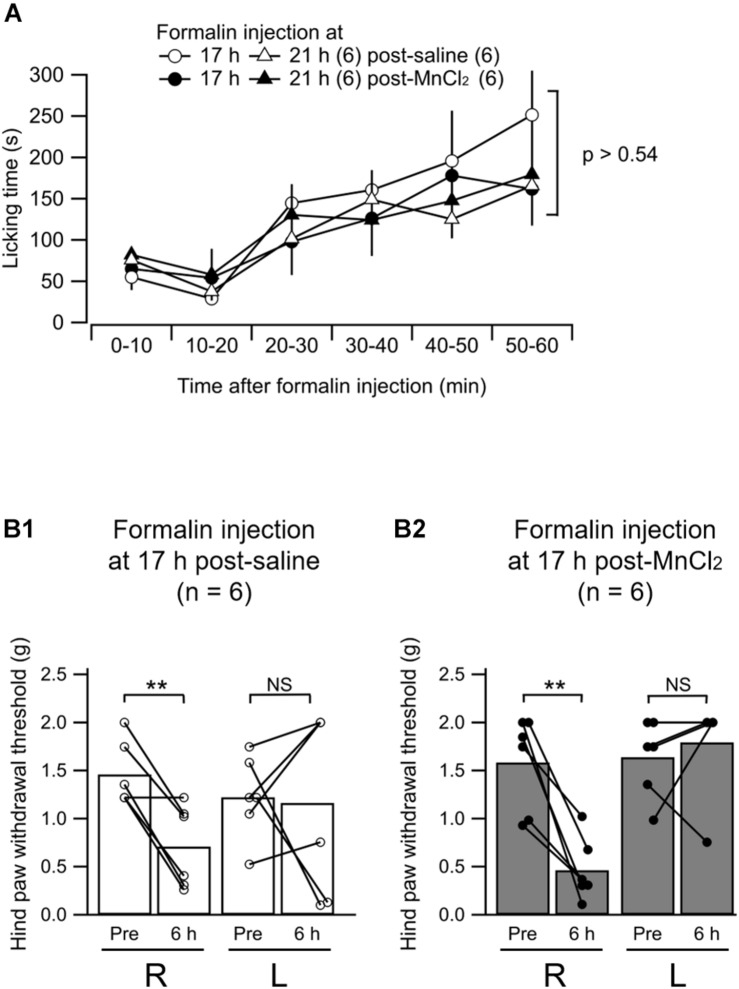
Formalin-induced nociceptive behavior. **(A)** Summary of the time course of the licking behavior after intraplantar injection of formalin (5%) in to the left hind paw. The open and filled markers indicate the responses recorded in mice with intravenous administration of saline and MnCl_2_, respectively. Circles, 17 h after administration (post-saline, *n* = 6; post-MnCl_2_, *n* = 6); triangles, 21 h after administration, (post-saline, *n* = 6; post-MnCl_2_, *n* = 6). Mean ± SEM. ANOVA followed by *post hoc* Gabriel’s test. Numbers in parentheses indicate the number of mice. **(B1,B2)** Summary of the paw withdrawal threshold as measured with von Frey filaments at 6 h after intraplantar injection of saline **(B1)** or formalin **(B2)** to the left hind paw. R, values from the right hind paw; L, values from the left. Pre, measurement at 2 h before intraplantar injection; 6 h, that at 6 h after injection. The bars show the mean and the markers show the values from each mouse. *P* < 0.005; NS, not significantly different; paired *t*-test.

We asked whether the MnCl_2_ injection in order to perform MEMRI affects the nociceptive behaviors of mice. First, we evaluated the spontaneous behavior typically observed following intraplantar injection of formalin. The animal receiving formalin showed spontaneous licking of the injected hind limb for more than 1 h after formalin injection (Figure 7A). This post-formalin behavior was similarly observed without any significant difference between the saline- and MnCl_2_-injected animals regardless of the time after MnCl_2_ injection at 17- and 21 h before formalin (Figure 7A).

We have already demonstrated that the mice that received unilateral hind paw injection of formalin show decrease in mechanical tactile threshold not only in the inflamed paw but also, rather more potently, in the contralateral paw at 6 h post-injection (Shinohara et al., 2017). We confirmed this behavioral change in mice with intraplantar formalin injection at 6 h post-injection at 17 h post-MnCl_2_ injection (Figures 7B1,B2). The paw withdrawal threshold in the hind limb contralateral to the formalin injection decreased significantly at 6 h post-formalin in both saline and MnCl_2_ injected animals 17 h before the formalin injection. This indicates that central sensitization underlying this contralateral hyperalgesia is not affected by the MnCl_2_ infusion. We failed to find a significant decrease in paw withdrawal threshold in the ipsilateral hind paw to the formalin injection presumably because of the severe edema in the injected paw (Figures 7B1,B2). This was observed in both saline-injected and MnCl_2_-injected animals (Figures 7B1,B2). These results indicate that prior injection of MnCl_2_ does not significantly affect the spontaneous and evoked nocifensive behaviors in the animals that received intraplantar formalin injection.

## Discussion

### General

By combining MEMRI in a 9.4-T high-field scanner and DREADD technologies, we have demonstrated, for the first time, that (1) widely distributed brain regions are activated between 2- and 6 h after the beginning of the inflammation, and (2) activation of a subset of these regions is dependent at least on the activation in the right amygdala. The most important and original aspects of these findings are that these activations were detected in spontaneously moving anesthetic-free animals with inflammatory pain.

### The Latent Chronification Model of Formalin Inflammatory Pain

We used intraplantar injection of formalin to trace shifts from acute to chronic pain. In a large body of pain research, this “formalin” model is used to evaluate phase I and II nocifensive behaviors such as flinching of the injected limb or licking of the injected paw appearing and terminating within ∼60 min after injection. It is generally acknowledged that these two phases correspond to the acute nociceptive and early inflammatory processes, respectively. We observed these typical early nocifensive responses lasting for ∼60 min in all mice receiving formalin injections but in none receiving saline injections. These behaviors were accompanied by clear signs of paw swelling in all mice administered formalin. Conventionally, these formalin-induced nocifensive behaviors are attributed to the acute central sensitization within the dorsal horn (Tjølsen et al., 1992) and this “formalin test” has been used to evaluate the effects of analgesics, for example, on the acute inflammatory pain at the spinal level (Barrot, 2012). For these reasons, we hereafter call this phase the “early nociceptive/inflammatory phase.”

Nonetheless, recently accumulated lines of evidence indicate that, after the fading of these acute nocifensive responses within ∼60 min, the brain structures involved in the establishment of chronic pain remain activated. Such activation includes increased pERK expression in the right CeA at 3 h post-formalin (Carrasquillo and Gereau, 2007) and augmented synaptic potentiation in the CeA at 6–24 h (Sugimura et al., 2016; Shinohara et al., 2017), accompanied by mechanical allodynia in both the inflamed and contralateral hind paws, a sign of central sensitization (Carrasquillo and Gereau, 2007; Shinohara et al., 2017). These latent changes are suggestive of plastic changes in the brain network underlying sensory and emotional aspects of pain, which finally lead to neuropathic pain-like phenotypes after 1–2 weeks post-formalin (Salinas-Abarca et al., 2017). In particular, this latent phase is characterized by right side-specific activation of the CeA, as evidenced by increased pERK expression (Carrasquillo and Gereau, 2007) and c-Fos expression (Miyazawa et al., 2018) observed at 3 h post-formalin. This strongly suggests that plastic changes in the brain would occur several hours after the initiation of the inflammation to cause a drastic shift from acute pain to chronic pain in these early few hours. On the basis of these recent findings, we hereafter name this phase the “cerebral sensitization phase.”

Because the objective of this study was to analyze the brain mechanism underlying the early establishment process of chronic pan, we focused on the changes in the brain activity in the cerebral sensitization phase at 2–6 h post-formalin. For time-differential analyses of Mn^2+^ accumulation, we scanned the brain in separate cohorts of mice immediately after the 2- and 6-h period of spontaneous free behavior after the formalin injection (Figure 1A). As mentioned above, the 2-h period was characterized by acute and intensive nocifensive hind limb movements lasting 60–90 min for all formalin-injected animals. After this 2-h period until the 6 h post-formalin (i.e., in the subsequent 4-h period), the animal behavior was characterized as follows: (1) no typical signs of acute nocifensive behaviors, unlike in the phase I and II period; (2) evident edema in the hind paw, suggesting that inflammatory signals remain elevated; (3) the animals were mostly awake and seemingly ate and drank as normal (however, the amount eaten and drunk was not evaluated); and (4) there was no specific interaction (e.g., repeated fighting and attacking) between the mice kept in the same home cage. It is thus expected that the changes in the brain manganese accumulation during this period might primarily represent the activities underlying the spontaneous pain, particularly during the early shift from the acute pain to chronic centralized pain.

### Advantages of Differential Manganese Visualization in Non-anesthetized Animals

#### MEMRI and fMRI

The principal strategy that we used to identify the brain regions specifically accumulating Mn^2+^ during chronic pain development was a statistical comparison of the values at the same voxel between the distinct cohorts. This was used to demonstrate (1) the brain regions activated within 2–6 h post-formalin (time-differential comparison) and (2) the effect of repeated activation of inhibitory DREADDs (group-differential comparison). Another approach to trace the process of cerebral sensitization would be to continuously analyze the recorded values of the same voxels from single animals using fMRI, which is suitable for detecting rapid time-dependent changes in BOLD signals or for comparing the statistical structures of the network organization in resting-state animals (Yee et al., 2015; Chang et al., 2016; Komaki et al., 2016). However, this approach is not suitable for comparisons between distinct animal cohorts (Jeong et al., 2016) or between remote time points (more than a few hours). In addition, fMRI scanning in animals requires anesthesia to avoid pain-associated escape behaviors, which disturbs pain-related brain activities, particularly those associated with spontaneous pain, or intensive training (Yee et al., 2015). Scanning without anesthesia has been successful for continuous recording within minutes (Chang et al., 2016), which is just at the limit for estimation of the resting-state functional connectivity. In contrast, MEMRI can visualize the neuronal activity of experimental animals during the unanesthetized state after MnCl_2_ administration, even they are under anesthesia when they are scanned by MEMRI. This is highly advantageous in analyzing spontaneous brain activities in freely moving awake animals without anesthesia, such as those in animals with chronic pain.

#### ROI-Based and Voxel-Wise Comparisons

Manganese-enhanced magnetic resonance imaging has been successfully used to analyze the influence of specific treatments on neuronal activities, which is based on comparisons between distinct cohorts of animals undergoing distinct treatments. The following two statistical approaches for differential analyses have been used: (1) region-of-interest (ROI)-based comparisons, and (2) voxel-wise comparisons (Devonshire et al., 2017). ROI-based comparisons depend on the summation of the voxel values within the defined brain regions and are suitable for evaluation of the activity within already defined brain regions. Multiple ROIs can be manually and visually defined in each animal, making statistical comparisons between cohorts easier and more reliable. This approach is particularly suitable for analyzing the data acquired with relatively low-field (<7 T) scanners (Jeong et al., 2016; Devonshire et al., 2017). However, because the ROI definition requires *a priori* knowledge of the brain regions hypothetically postulated to be activated. This approach is not powerful enough for revealing “undescribed” sets of regions that would show altered activities under distinct conditions. In contrast, voxel-wise comparisons, which we used in this study, enable such undescribed differences to be revealed.

Interestingly, Devonshire et al. (2017) used both ROI-based and voxel-wise approaches to uncover the difference in Mn^2+^ accumulation in rats and failed, against their expectations, to observe any significant differences between saline and acute or chronic pain models with either of these two approaches. These negative results, unlike ours, might be attributed partly to the lower resolution (a 7-T magnet with a 30-cm bore without any ultra-low temperature cryogenic probe) of the MEMRI and also to a long MnCl_2_ application period (>7 days) for chronic pain models. Because we used a 9.4-T magnet combined with a cryogenic probe allowing a 100-μm voxel size, the complications inherent to the registration process (i.e., rectification and standardization of the acquired images in each animal to a defined brain “template”) would have been minimized. Given that we examined the regions of the brain that are more activated during the shift from direct nociceptive pain to that with central sensitization (Carrasquillo and Gereau, 2007; Shinohara et al., 2017; Miyazawa et al., 2018) and which regions are inhibited by suppressing the neuronal excitation in the right amygdala with DREADD technology during the course of this shift, we chose to use voxel-wise analysis and successfully obtained significant differences in these comparisons.

### Regions Activated Between 2 and 6 h Post-formalin

Conceptually, the Mn^2+^ accumulation at 6 h post-formalin is a sum of the following components: (1) Mn^2+^ accumulation within the early nociceptive/inflammatory phase, (2) that spanning over this 6-h period with sustained activity, and (3) that within the cerebral sensitization phase. Because our goal was to identify the brain areas specifically activated in the cerebral sensitization phase (i.e., component 3), we visualized the Mn^2+^ accumulation from 2 h to 6 h post-formalin by comparing the images from each cohort (Figure 2B). We suggest that these widely spread brain regions showing significantly larger signals in [6 h Formalin] than in [2 h Formalin] would partly underlie the cerebral sensitization. These regions contained: somatosensation-associated regions (the right primary/secondary somatosensory cortex and bilateral thalamus), limbic regions associated with survival valence and/or salience (i.e., emotion and rewards: the right insula cortex, right nucleus accumbens, bilateral caudate putamen, right amygdala, and left ventral tegmental area), and regions more involved in other functions (the right globus pallidus and bilateral substantia nigra). This pattern of activation is globally but not exactly in agreement with previous reports in human patients that analyzed brain activity in awake patients with chronic spontaneous pain using BOLD signals (Hashmi et al., 2013; Vachon-Presseau et al., 2016) and PET (Kulkarni et al., 2007) and also in lightly anesthetized rodents with experimental nerve injury (Baliki et al., 2014). This result supports the idea that chronic pain is accompanied by “large-scale brain functional and morphological re-organization” in various species (Baliki et al., 2014).

As expected, the regions showing higher Mn^2+^ signal in [6 h Formalin] than in [6 h Saline] (Figure 6D) were more extensive than the regions with larger Mn^2+^ signal in [6 h Formalin] than in [2 h Formalin] (Figure 2B). This result was expected because the difference between [6 h Formalin] than in [6 h Saline] corresponds to the sum of the three components listed above, whereas the difference between [6 h Formalin] and [2 h Formalin] roughly corresponds only to component 3. The regions showing a significant difference between [6 h Formalin] and [6 h Saline] but not between [6 h Formalin] and [2 h Formalin] included the primary/secondary motor cortex, auditory cortex, visual cortex, anterior cingulate cortex, hypothalamus, and hippocampus. It is interpreted that these structures belong to the component 2 activation that shows general and sustained activation after the initial nociception/inflammation until at least 6 h post-formalin. The anterior cingulate cortex is repeatedly reported to be activated by acute, on-going nociception as well as pain evoked by stimulation (Kulkarni et al., 2007). However, the mechanisms of the activation in the other areas need to be clarified.

On the other hand, we failed to identify regions with a significantly larger signal in the formalin group than in the saline group at 2 h (Figure 6C). It is likely that the Mn^2+^ accumulation during the early nociceptive/inflammatory phase (component 1) was not sufficient to reach a significant difference. The shorter time duration of component 1 for Mn^2+^ accumulation (2 h) compared with components 2 and 3 (6- and 4 h, respectively) might also have influenced this result. Another interpretation would be that the extracellular Mn^2+^ concentration at 21 h post-MnCl_2_ injection, when [2 h formalin] and [2 h saline] groups were made, was smaller than that at 17 h, resulting in smaller uptake of Mn^2+^ into the neurons despite acute nocifensive behaviors, making the saline-formalin difference insignificant. It has been shown that the brain tissue Mn^2+^ concentration increases immediately after systemic injection of MnCl_2_ and reaches at the peak value around 24 h (Aoki et al., 2004). Though it remains undetermined whether there is an abrupt drop of Mn^2+^ concentration from 17 h to 21 h and then to 24 h, it would be more reasonable to assume that the intra-tissue Mn^2+^ concentration does not change largely in a few hours before and after 24 h post-Mn^2+^ injection because the relatively high Mn^2+^ level is maintained through the first week (Aoki et al., 2004). This possibility would require a more precise evaluation of the time-course of intracerebral Mn^2+^ concentration in future studies.

### The Role of the Amygdala and Transfected Structures in the Late-Phase Co-activation of Limbic Regions

The Mn^2+^ signal at 6 h post-formalin was significantly and robustly smaller in the amygdala after repeated intraperitoneal injection of CNO in the animals expressing hM4D(G_i_) receptors in the amygdala than those expressing EGFP only. This study is the first to demonstrate a significant change in the Mn^2+^ signal with MEMRI via chemogenetic manipulation of neuronal activities. This is important because this result provides direct evidence that the decreased neuronal excitability indeed decreases the Mn^2+^ accumulation. This is also in agreement with the idea that the Mn^2+^ signal indeed reflects Mn^2+^ entry through voltage-dependent Ca^2+^ channels and NMDA receptor channels activated in response to neuronal excitation(Silva and Bock, 2008; Kikuta et al., 2015). The most important and novel finding of this DREADD-MEMRI study is that the DREADD-mediated suppression of the amygdala and transfected structures decreased the Mn^2+^ signal not only in the amygdala but also in multiple regions remote from those structures expressing mCherry combined with hM4D receptors. These regions with decreased Mn^2+^ signals with CNO included the infralimbic cortex, nucleus accumbens, caudate putamen, globus pallidus, ventral tegmental area and substantia nigra. Many of these limbic and associated structures have been reported to be activated during spontaneous pain in chronic pain patients (Hashmi et al., 2013; Vachon-Presseau et al., 2016).

In contrast, the structures involved in sensory aspects of pain, such as the insular cortex, primary/secondary sensory cortex, and thalamus, did not show a significant reduction in Mn^2+^ signal after DREADD-mediated suppression of the amygdala and transfected structures. As the BLA and CeA consistently expressed mCherry, and therefore hM4D receptors, in most of the mice in which CNO was injected, the most straight-forward interpretation of this secondary suppression is that these structures – the nucleus accumbens, globus pallidus, ventral tegmental area, and substantia nigra – are downstream of the amygdala activity and the suppression of the amygdala activity resulted in reduced activity in these structures. Indeed, it has been shown that neurons in the BLA and/or CeA project to many of these structures (Pitkänen, 2000; Sah et al., 2003). Therefore, it might be likely that the suppression of these excitatory outputs from the amygdala and transfected structures would have played a major role in suppressing these other limbic regions.

However, some lines of evidence do not fit this simple interpretation. First, the CeA is mostly composed of GABAergic neurons. In particular, the outputs from the CeA, mostly arising from the medial part, target these limbic structures and are GABAergic. Second, we have previously reported a markedly larger number of c-Fos–positive neurons in the right CeA than in the right BLA 3 h after formalin injection into the facial regions in rats (Miyazawa et al., 2018). Third, in the same intraplantar formalin injection model of mice used in this study, we have observed a very similar synaptic potentiation at the synapses between the lateral parabrachial nucleus and the CeA neurons (Shinohara et al., 2017) to that which we observed in the rats with facial formalin injection (Miyazawa et al., 2018) at 6 h post-formalin. These lines of evidence indicate that the predominant excitation of the CeA, rather than the BLA, is more likely in this model. If this is the case, the decreased Mn^2+^ signal in these limbic structures by CNO would not be the simple consequence of the suppression of CeA inhibitory outputs. Importantly, these structures including the amygdala have mutual connections between each other (Pitkänen, 2000; Sah et al., 2003), forming a mutually interfering “web”-like network. Therefore, it is hypothetically interpreted that a dysfunction of the right amygdala with hM4D receptor activation might have caused a long-term change in the activity in the hubs, which resulted in a lower Mn^2+^ signal in the members of this mutually connected, functionally related, network hub. Such a slow and long-lasting mutual influence between distinct voxels cannot be detected through functional connectivity analysis of the fMRI, which simply calculates the cross-correlation coefficient of the two voxels over a short time of period. The present results indicate that the amygdala, infralimbic cortex, nucleus accumbens, caudate putamen, globus pallidus, ventral tegmental area and substantia nigra form a functional unitary web that requires the amygdala activation, at least, to be co-activated during the developing phase of the chronic inflammatory pain. This result is interesting because most of these nuclei share roles in representing the survival/defense valence of the on-going situation (Denk et al., 2014; LeDoux and Brown, 2017). In addition, it should be admitted that the chemogenetic approach we employed was region/structure-based transfection of DREADD receptors, indicating that the neurons located in that region (around the amygdala) were non-selectively affected by CNO injection regardless of their functions. Use of marker protein-dependent expression system using cre recombinase (Roth, 2016) and activity-dependent expression system using “Fos-TRAP” (Guenthner et al., 2013) and/or “CANE” (Sakurai et al., 2016) technologies would more selectively affect a specific set of neurons and provide insights on functional role of a specific set of neurons in pain chronification process. In this regard, the present study would provide the first example of the steps toward the wide application of DREADD technique to MEMRI-based analysis of mutual connectional causality of brain areas activated in association with chronic pain. In a separate study, we have used rats expressing cre recombinase under vesicular GABA transporter (VGAT) promotor (Soma et al., 2019) to have DREADDs expressed in inhibitory neurons (i.e., most neurons) in the CeA and found that pharmacological activation of the DREADDs in the right CeA potently modulates mechanosensitivity of the hind paw (Kato et al., 2016). This suggests that not only members of the limbic system but also the descending pain modulatory system is under the influence of the amygdala neurons. The changes in MEMRI signal we observed in the substructures of the limbic system might be mediated partly by such changes in the descending pain modulatory system after the suppression of the amygdala and surrounding structures. It is an interesting subject to be clarified in future studies.

Because we have manipulated the excitability of the neurons in the right amygdala with DREADD technique, it remains to be addressed (1) whether inhibition of the neurons in the left amygdala has similar consequences to what we have observed in this study with right amygdala inhibition and (2) what would be the consequences of suppressing the other structures than the amygdala. These questions are beyond the scope of this study and it is expected that these questions would be addressed with the DREADD-MEMRI approaches described here to various pain models.

## Conclusion

In this study, we used time-differential analysis of MEMRI to demonstrate, for the first time, elevated spontaneous activities in broad areas of the brain during the early developing “cerebral sensitization” phase of chronic pain with sustained inflammation. In addition, by combining DREADD technology with MEMRI (here named DREADD-MEMRI), we identified for the first time that a set of limbic regions requires sustained enhanced amygdala activity to be activated during this sustained inflammation. We speculate that these areas under amygdala influence form a functional unit playing roles essential for the “survival/defense valence” of chronic pain. DREADD-MEMRI is powerful enough to reveal such a functional association with slow spontaneous co-activation, which cannot be directly detected by functional connectivity analysis of BOLD signals. As biochemical changes, such as expression of phosphorylated ERK and c-Fos (Carrasquillo and Gereau, 2007; Miyazawa et al., 2018), and electrophysiological changes, such as synaptic potentiation (Shinohara et al., 2017; Miyazawa et al., 2018), are observed specifically in the right CeA in the latent phase of rodent formalin models, right CeA activation would be one of the key processes in the brain state transition from acute to chronic pain. In addition to these, the present findings using DREADD-MEMRI would add novel aspects of the information as to how the brain networks are sequentially activated during the course of chronic pain-associated changes.

## Data Availability

The datasets generated for this study are available on request to the corresponding author.

## Ethics Statement

Animal Subjects: The animal study was reviewed and approved by the Institutional Animal Care and Use Committee of the Jikei University.

## Author Contributions

DA and KS performed the MRI experiments. DA, KS, TT, and FK analyzed the MEMRI data. YT and YS performed the electrophysiological experiments. DA, YT, YS, and FK analyzed the electrophysiological data. YS and MS designed the AAV transfection experiments. DA, YT, and FK analyzed the histology data. FK and KM conceptualized and realized the experimental designs. All authors participated in discussing the data, and read and approved the final manuscript.

## Conflict of Interest Statement

The authors declare that the research was conducted in the absence of any commercial or financial relationships that could be construed as a potential conflict of interest.

## Footnotes

1 http://people.cas.sc.edu/rorden/mricro/bru2anz/index.html

2 https://www.nitrc.org/projects/mricron
