# Supplementary material for: Primary Role of the Amygdala in Spontaneous Inflammatory Pain- Associated Activation of Pain Networks – A Chemogenetic Manganese-Enhanced MRI Approach
Source: Front Neural Circuits. 2019 Oct 1;13:58. doi: 10.3389/fncir.2019.00058 (PMC6779784; doi:10.3389/fncir.2019.00058)
Supplement: Supplementary file 1 [file Data_Sheet_1.pdf]

Figure S1_1A

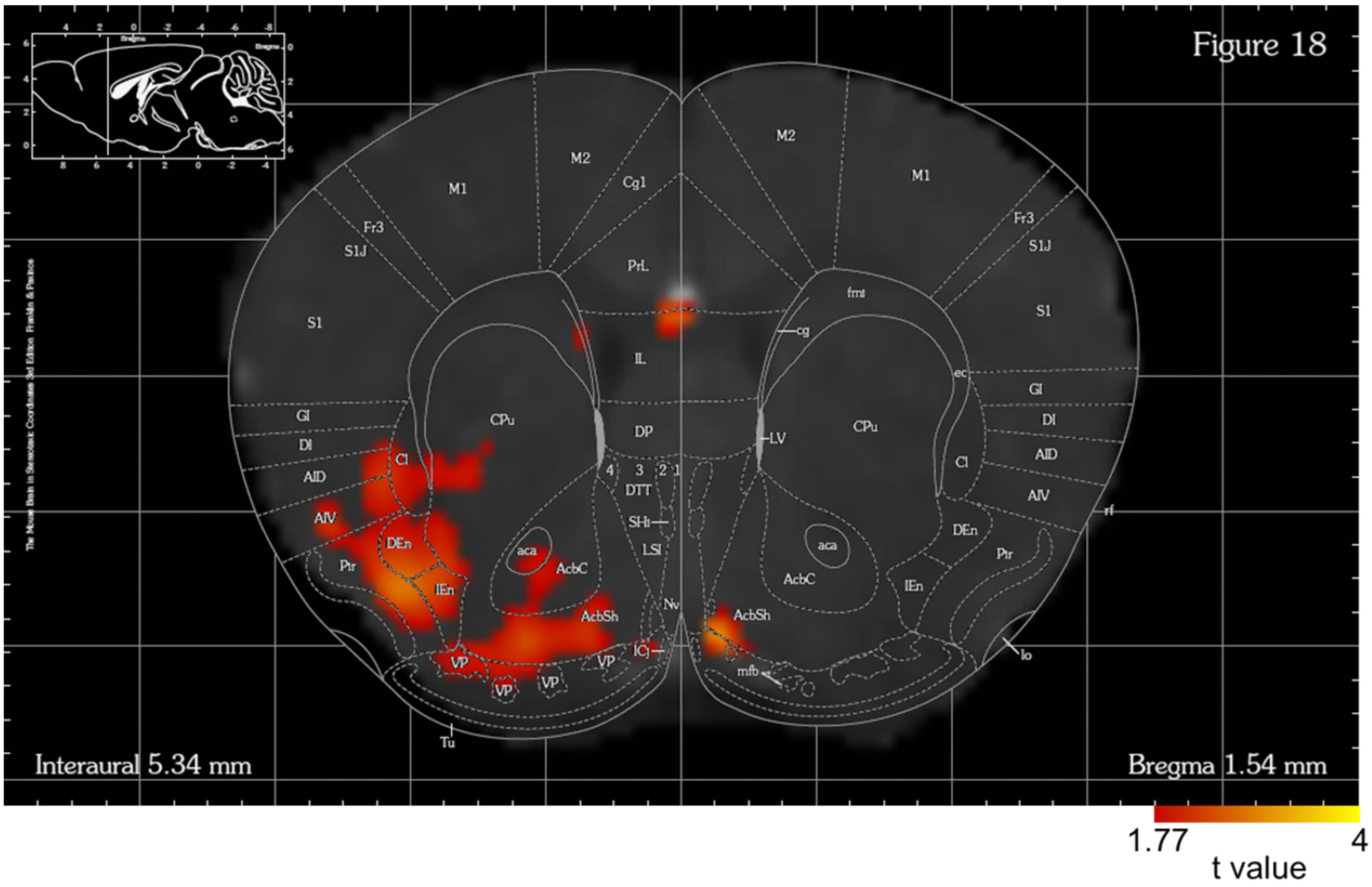

Figure S1_1B

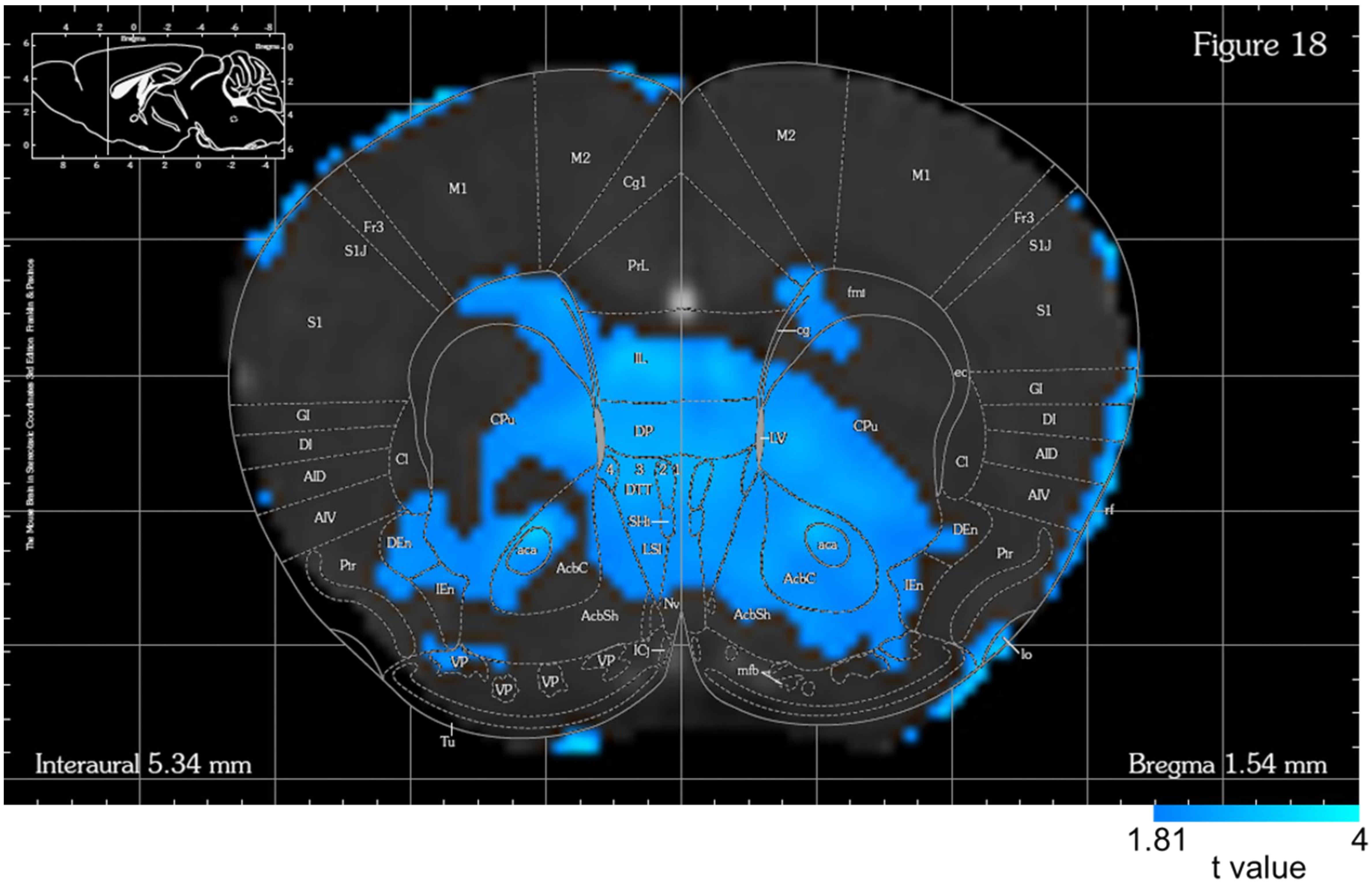

Figure S1_2A

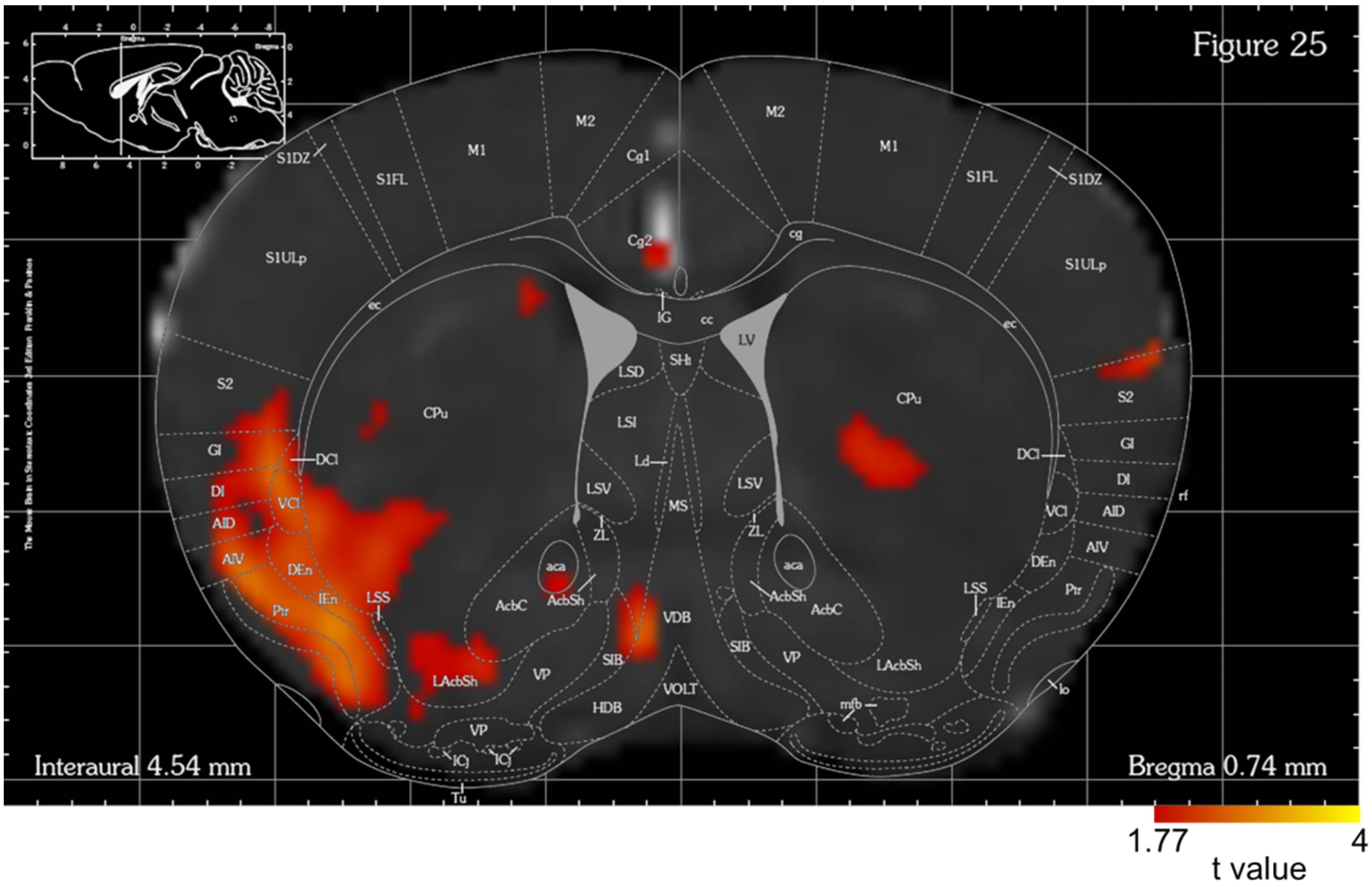

Figure S1_2B

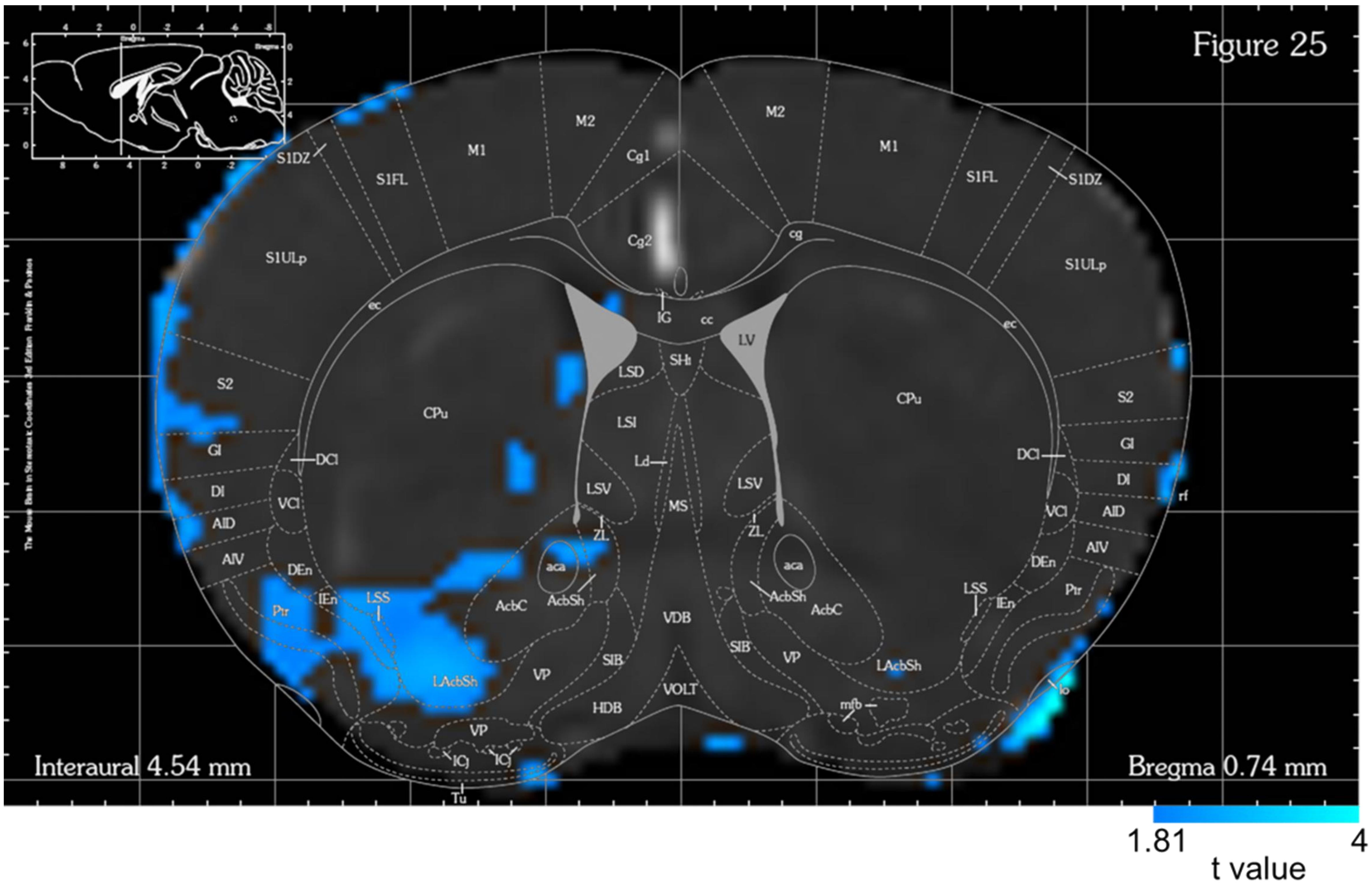

Figure S1_3A

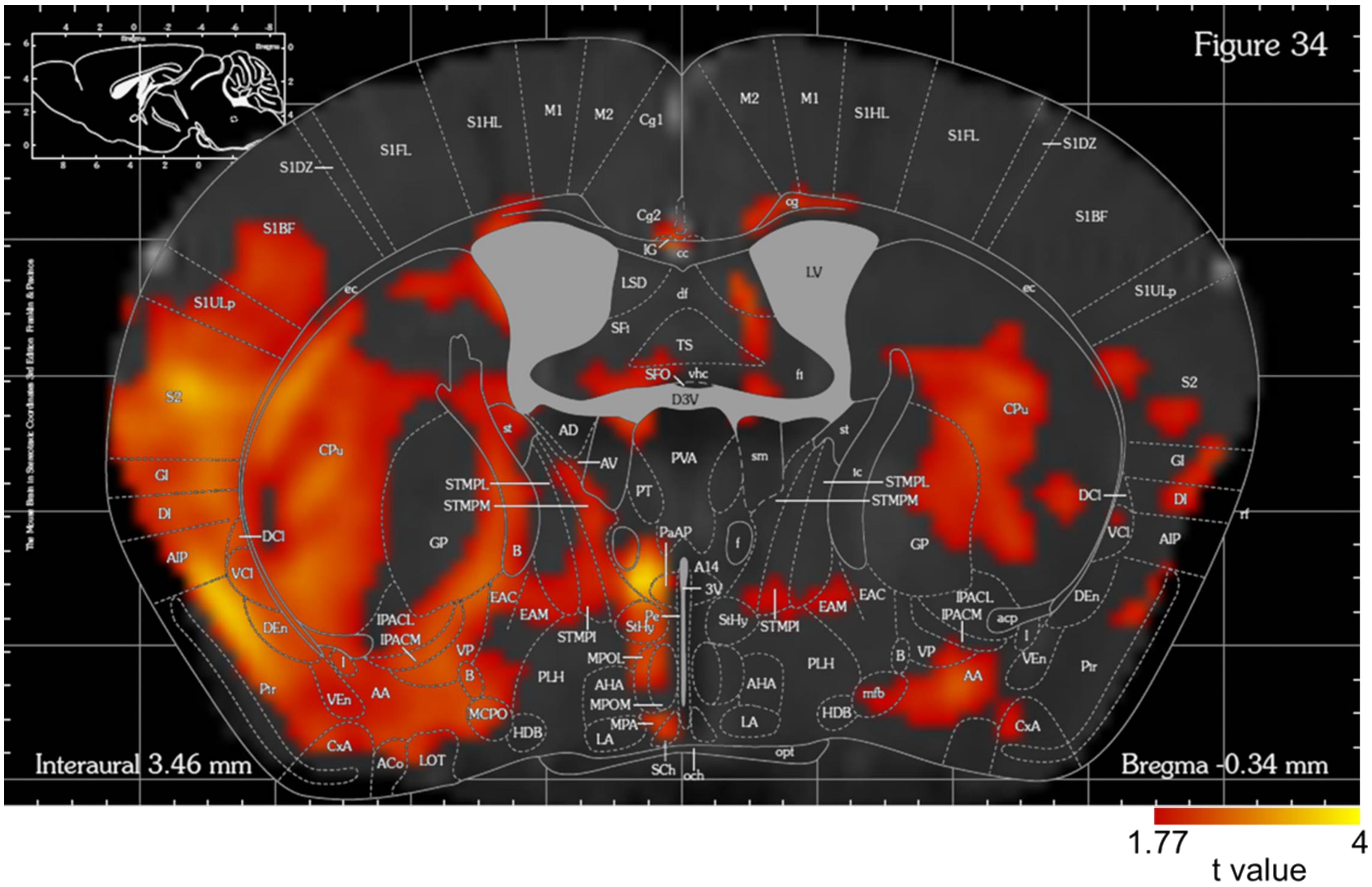

Figure S1_3B

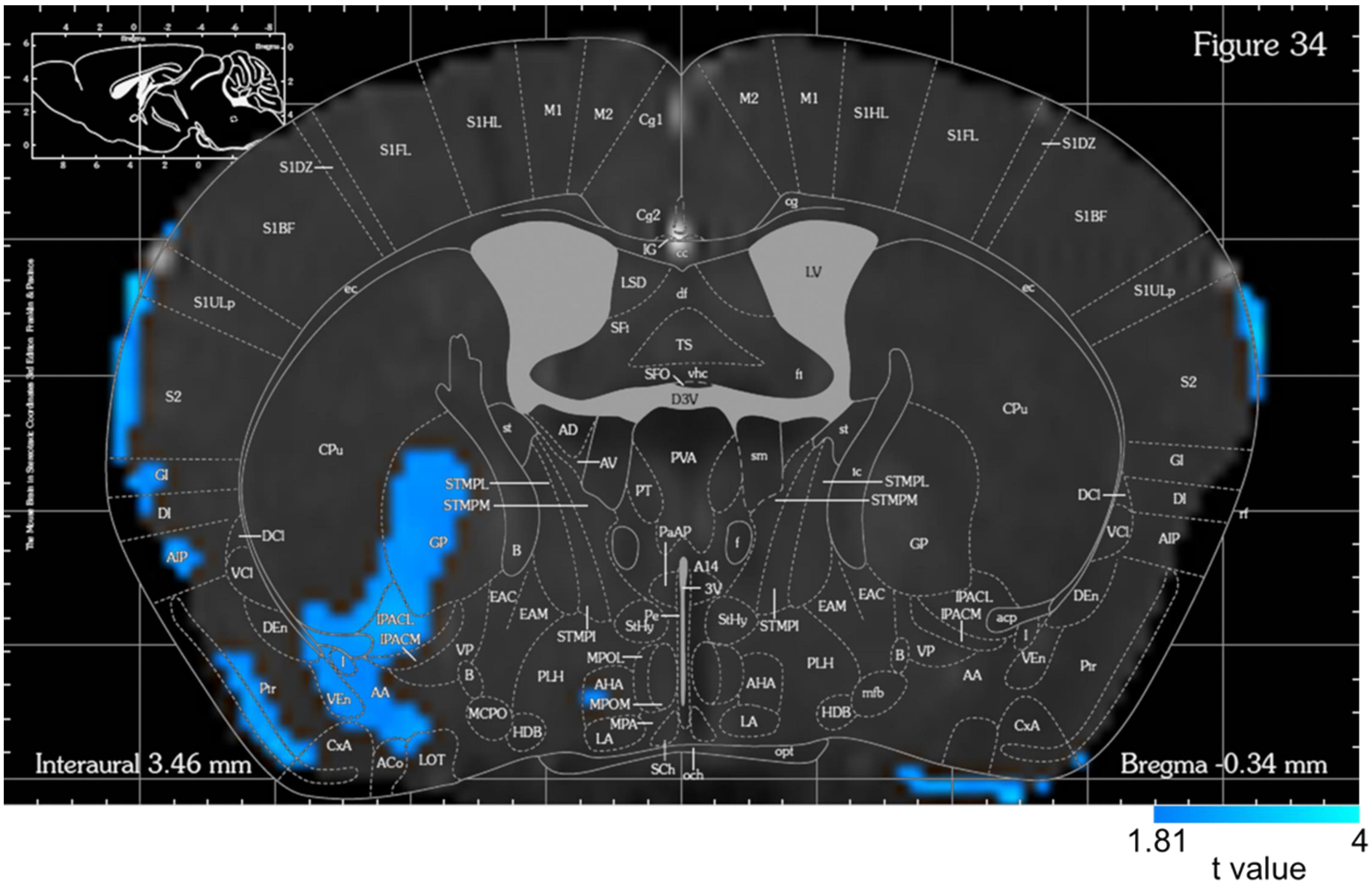

Figure S1_4A

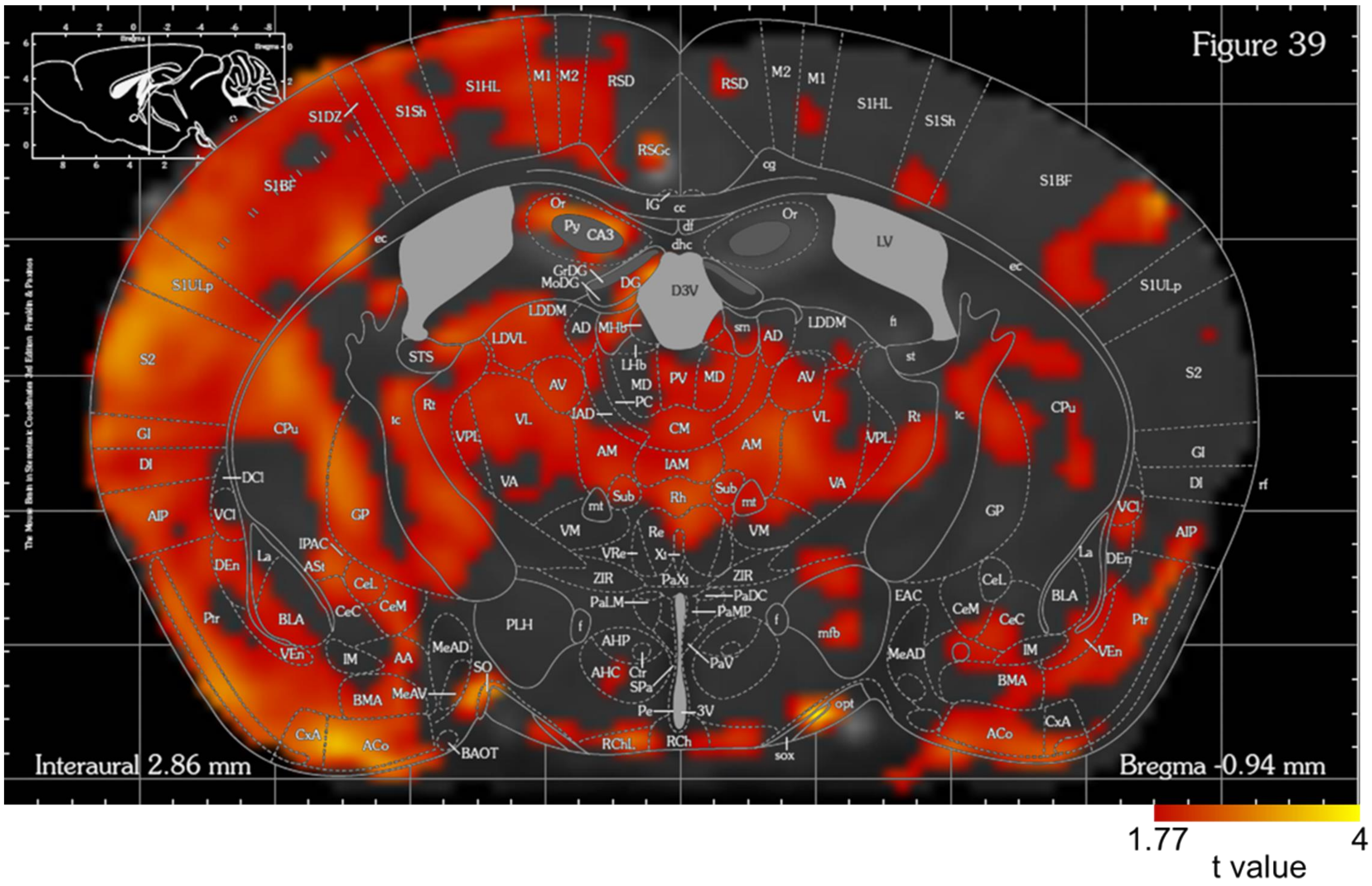

Figure S1_4B

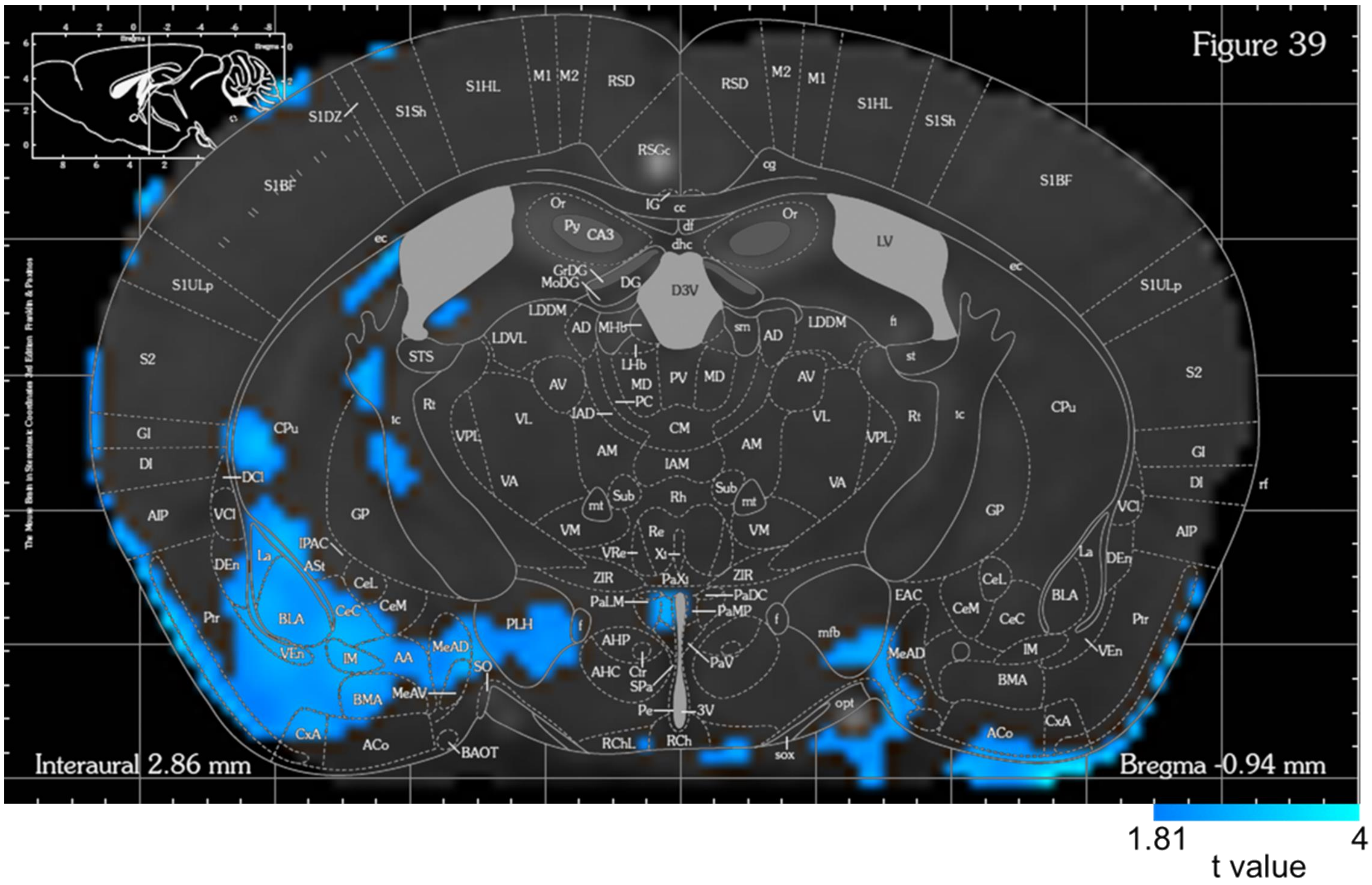

Figure S1_5A

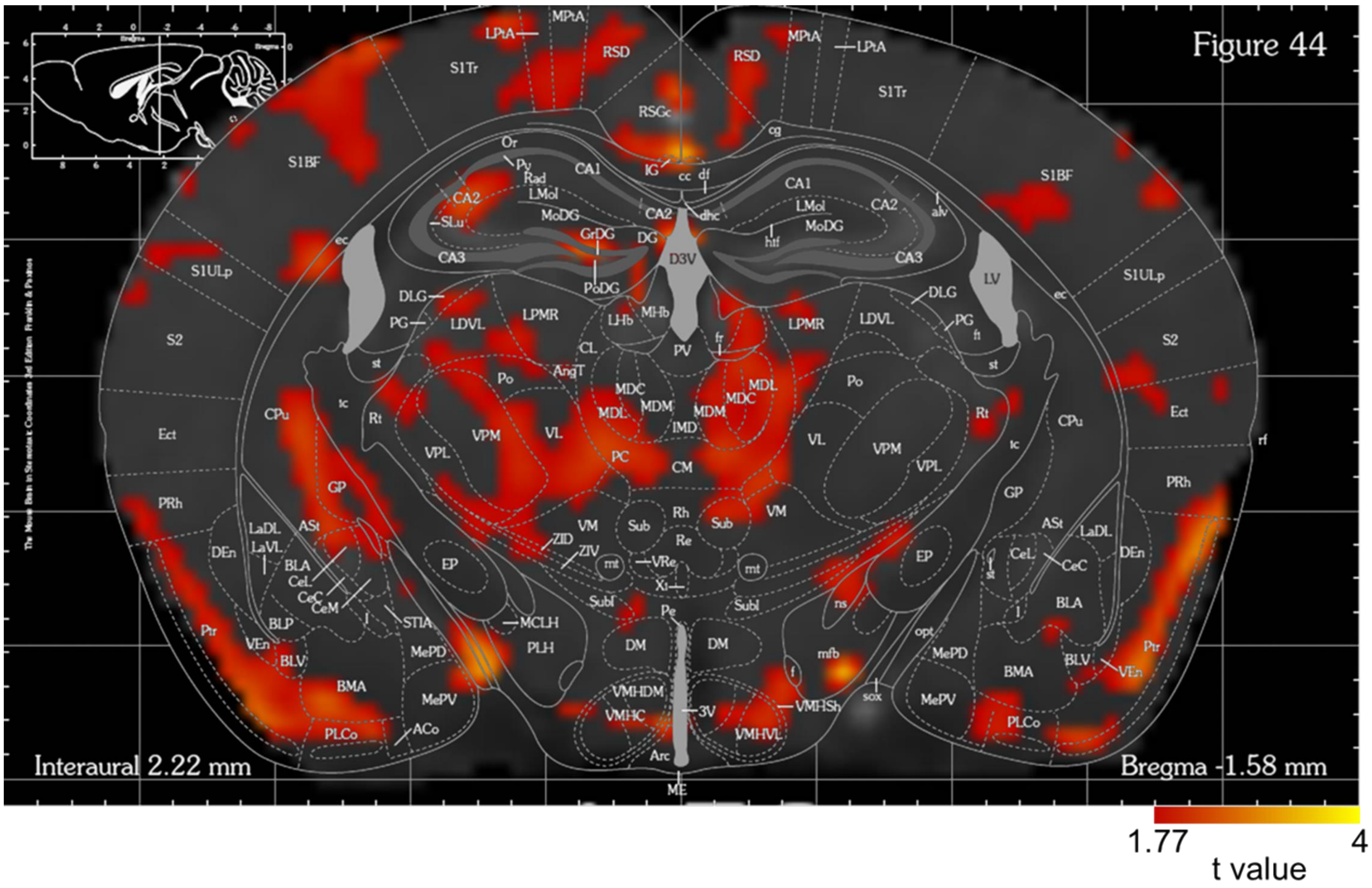

Figure S1_5B

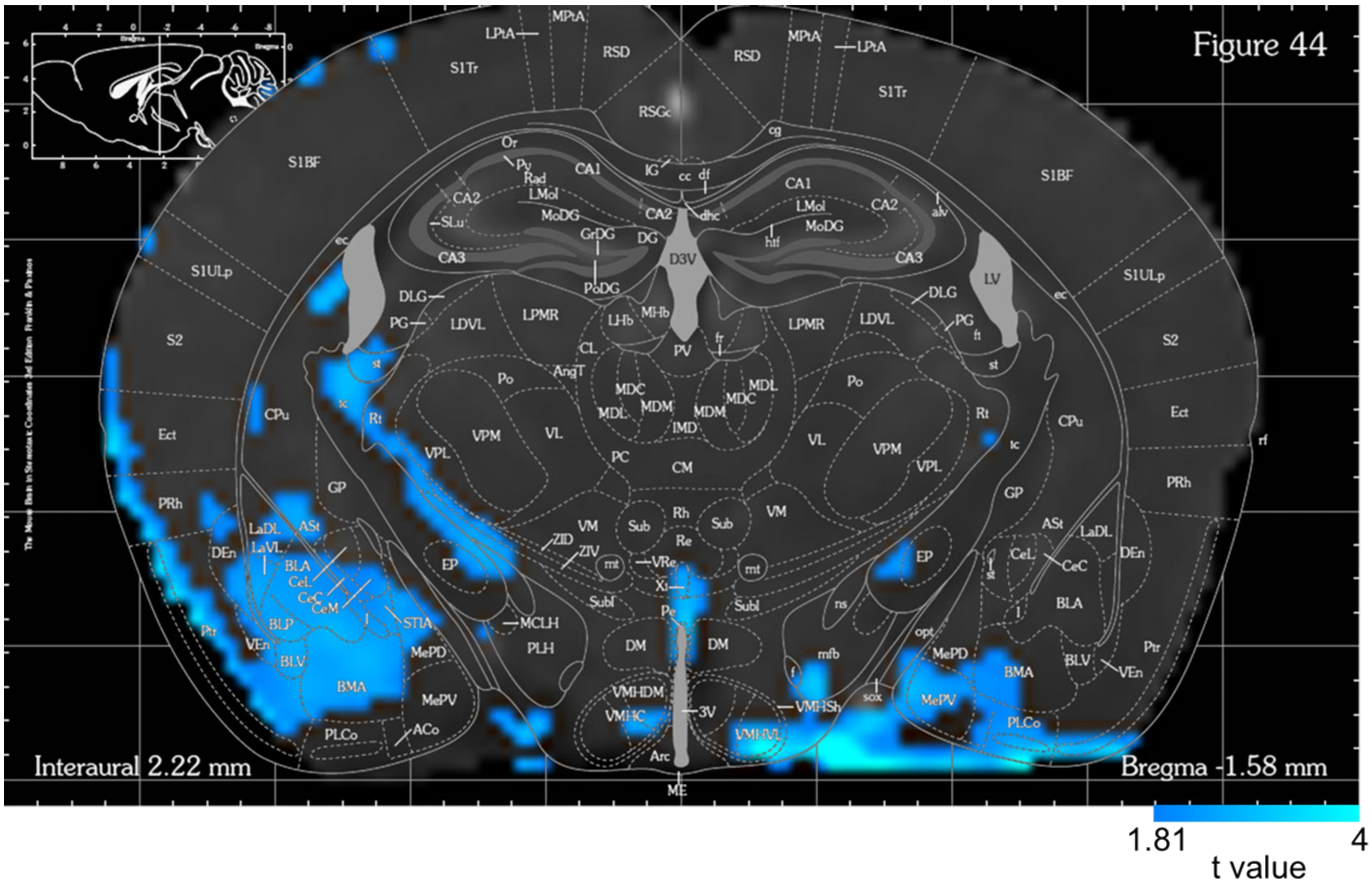

Figure S1_6A

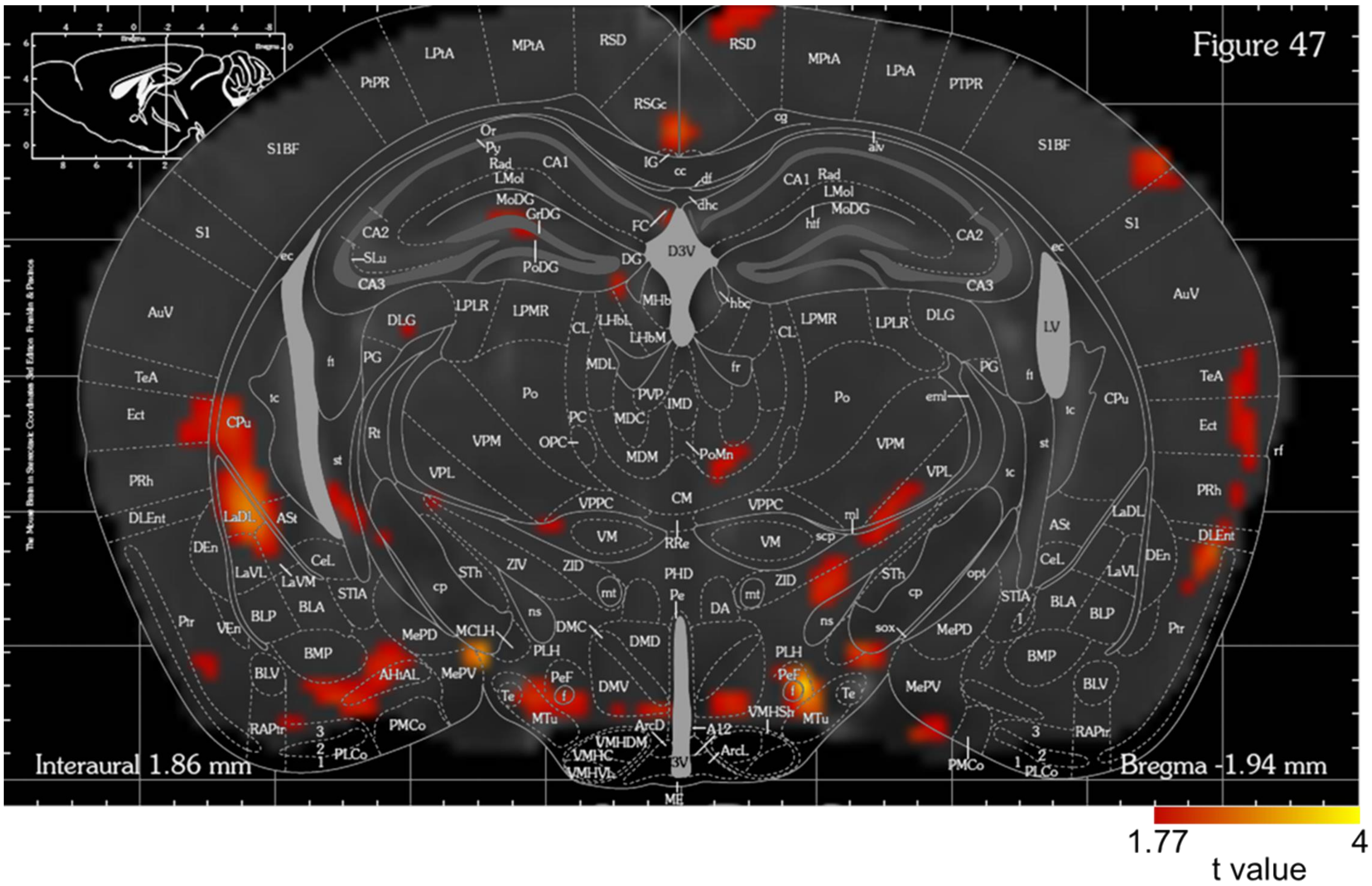

Figure S1_6B

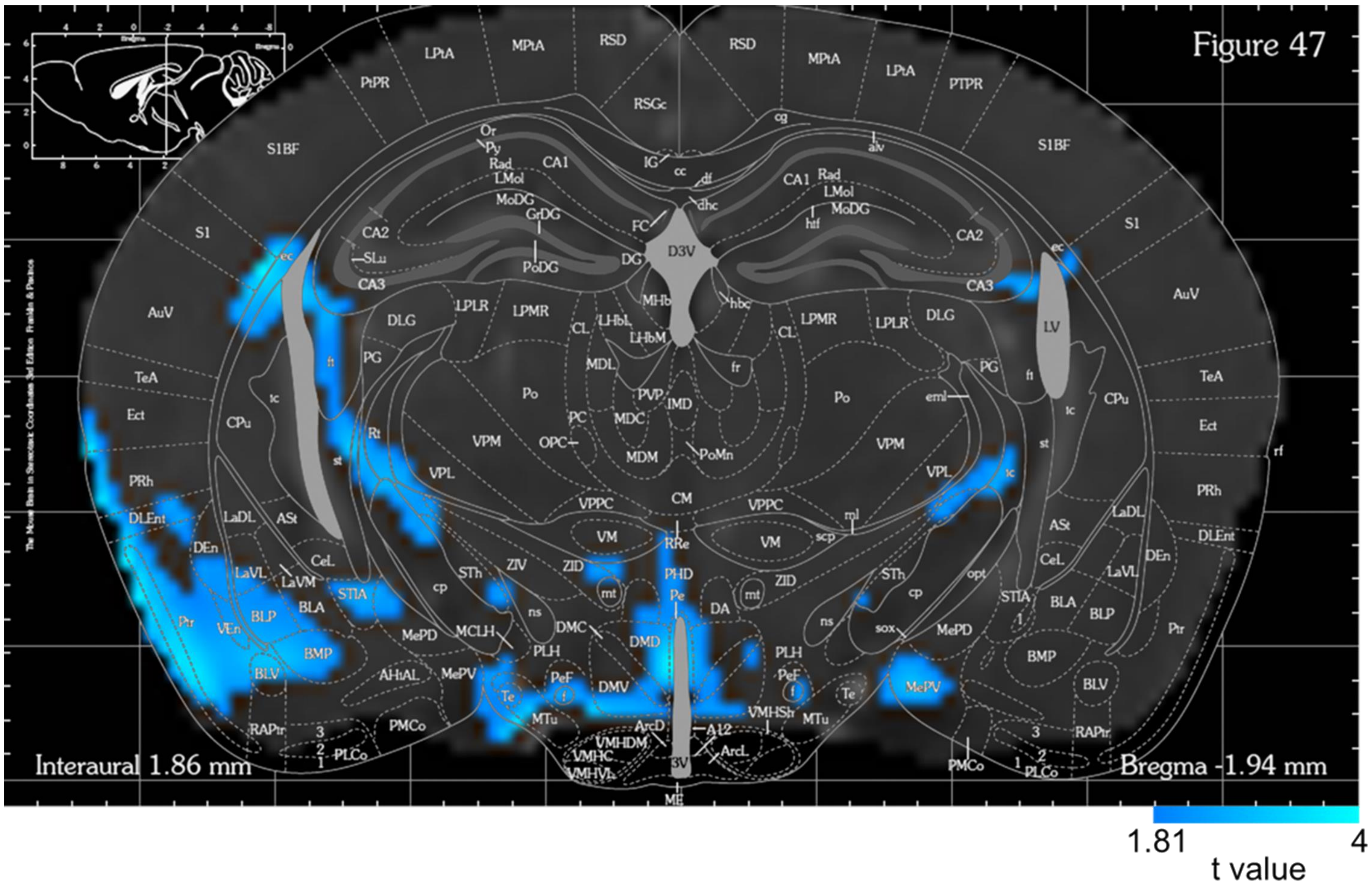

Figure S1_7A

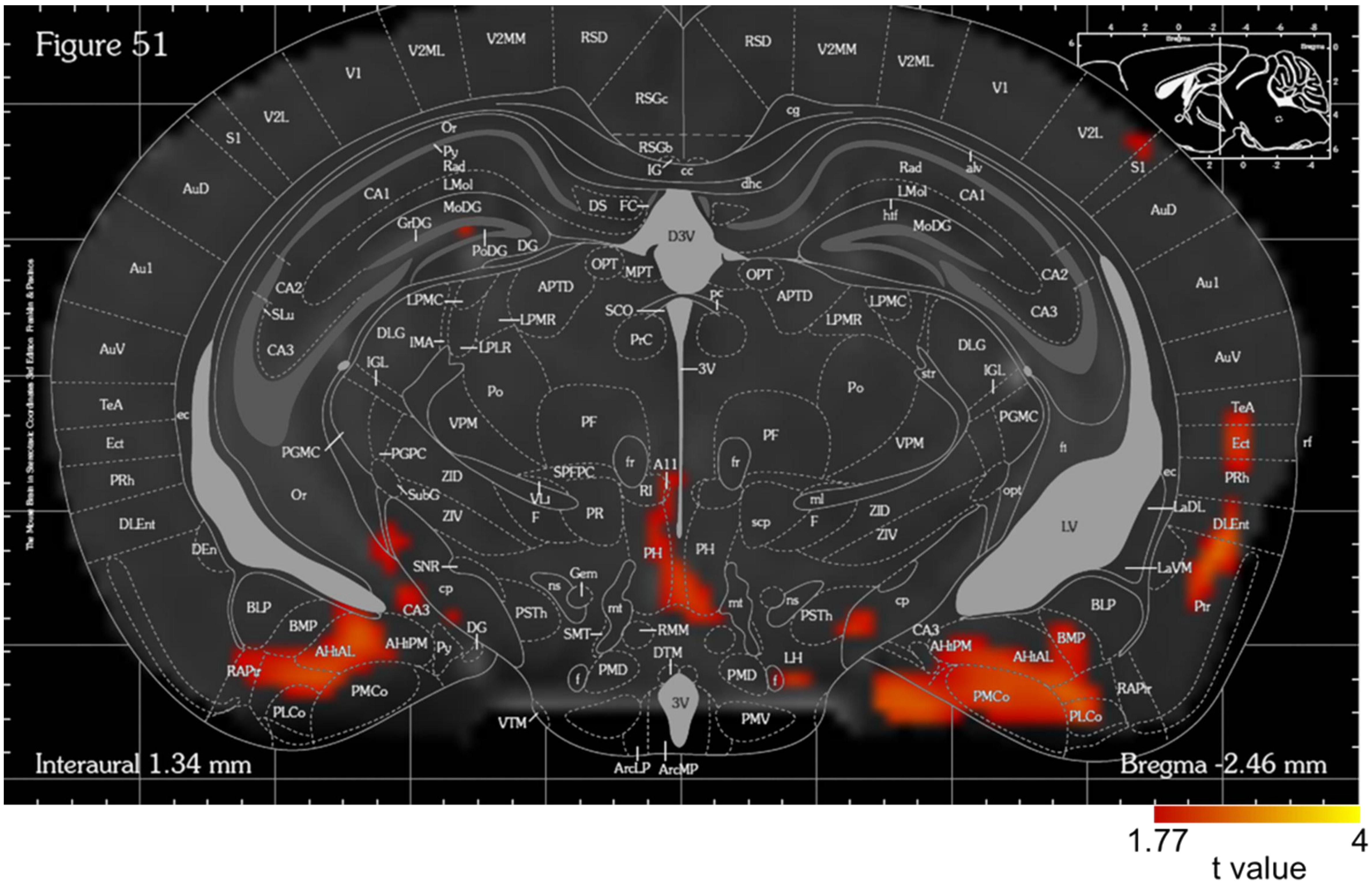

Figure S1_7B

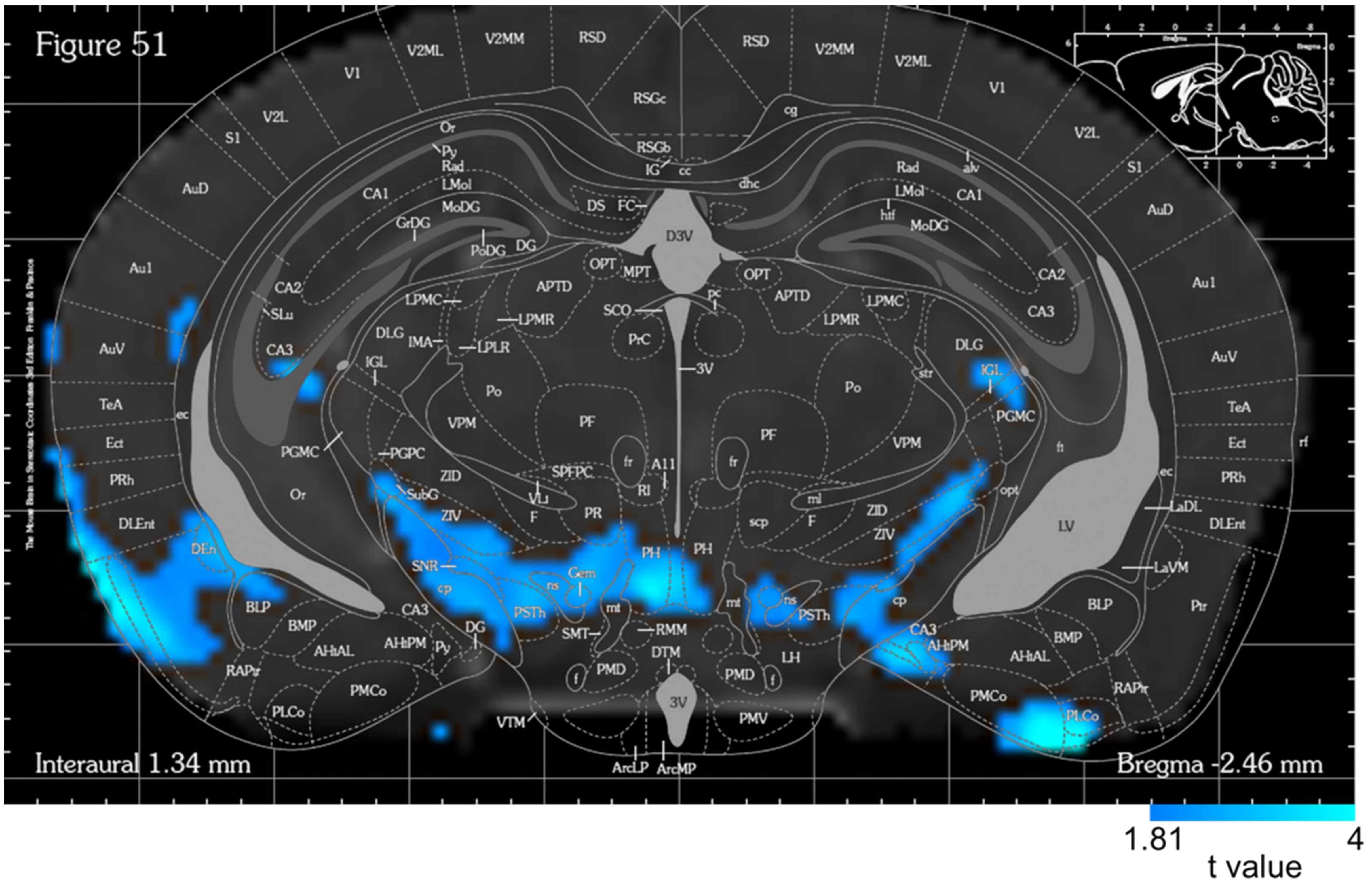

Figure S1_8A

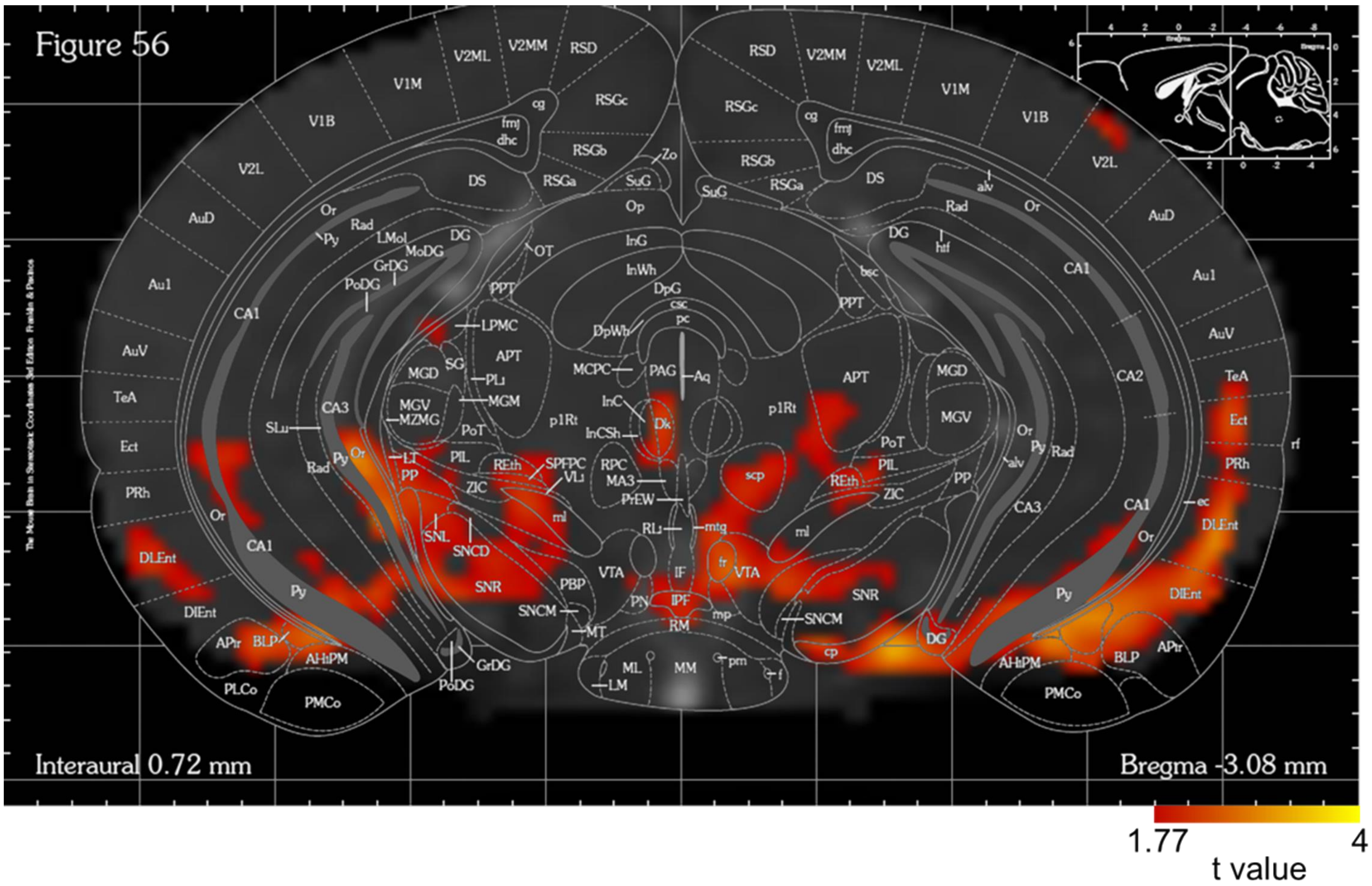

Figure S1_8B

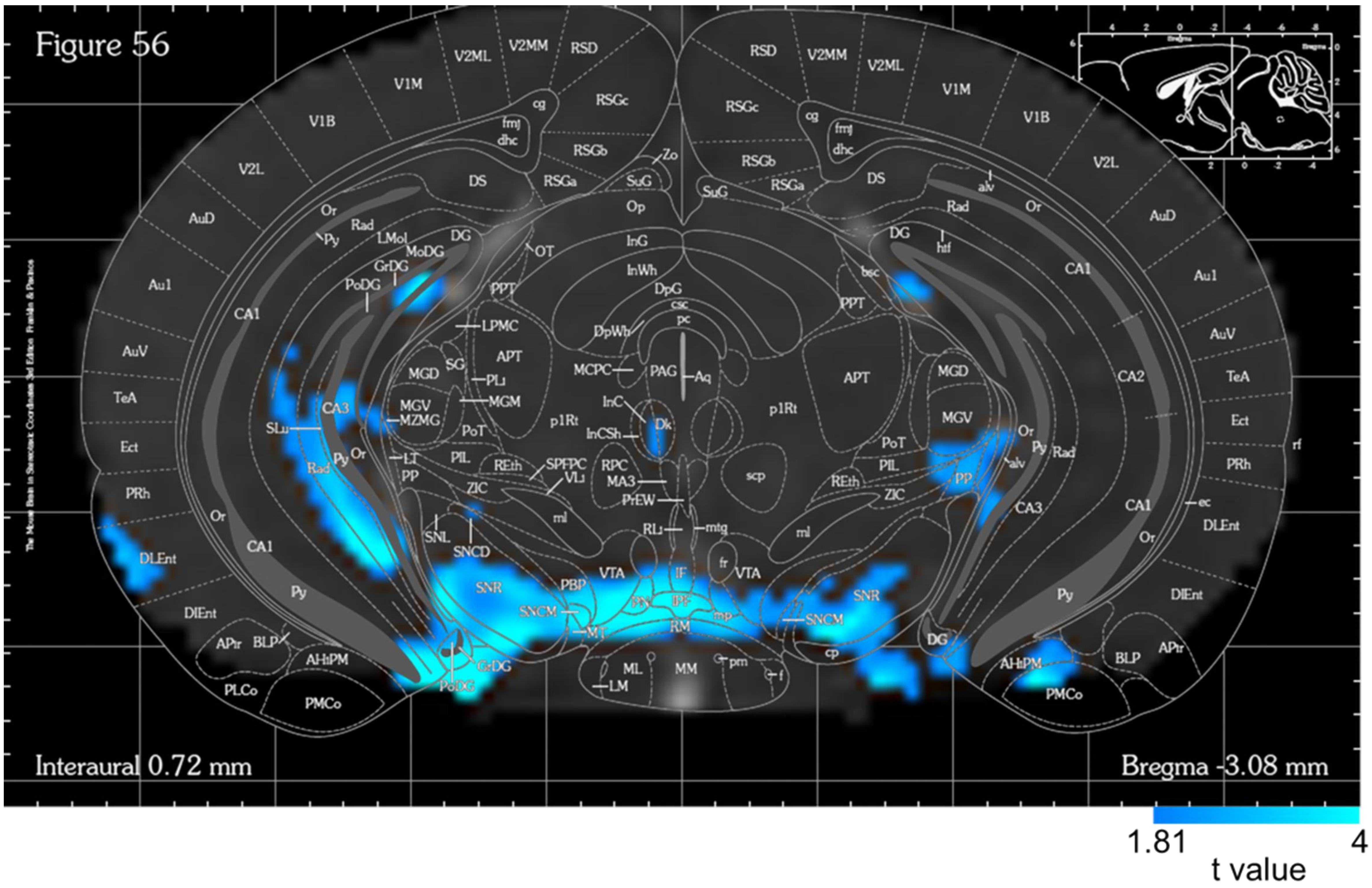

Supplementary Material

1 Supplementary Figures

Supplementary Figure 1. Merged images of the T-value plots and Franklin and Paxinos mouse brain atlas. T value is overlaid to the normalized brain template and corresponding Franklin and Paxinos mouse brain atlas (Franklin and Paxinos, 2008). S1_1A (bregma 1.54), S1_2A (bregma 0.74), S1_3A (bregma –0.34), S1_4A (bregma –0.94), S1_5A (bregma –1.58), S1_6A (bregma –1.94), S1_7A (bregma –2.46), S1_8A (bregma –3.08) are typical images showing the signal increase in [6 h Formalin] compared to [2 h Formalin]. S1_1B (bregma 1.54), S1_2B (bregma 0.74), S1-3B (bregma –0.34), S1_4B (bregma –0.94), S1_5B (bregma –1.58), S1_6B (bregma –1.94), S1_7B (bregma –2.46), S1_8B (bregma –3.08) are typical images showing the signal decrease in [hM4D] compared to [EGFP]. [The drawings and schema in this figure are partially taken from the atlas by Franklin KBJ & Paxinos G (2008), used with permission. These were published in "The mouse brain in stereotaxic coordinates, Third Edition (compact version)", Franklin KBJ & Paxinos G, Copyright Elsevier (2008).]
